# 2,2′-Bipyridine Derivatives as Halogen Bond Acceptors in Multicomponent Crystals

**DOI:** 10.1021/acs.cgd.3c01055

**Published:** 2023-11-09

**Authors:** Filip Kučas, Lidija Posavec, Vinko Nemec, Nikola Bedeković, Dominik Cinčić

**Affiliations:** † Department of Chemistry, Faculty of Science, University of Zagreb, Horvatovac 102a, 10000 Zagreb, Croatia

## Abstract

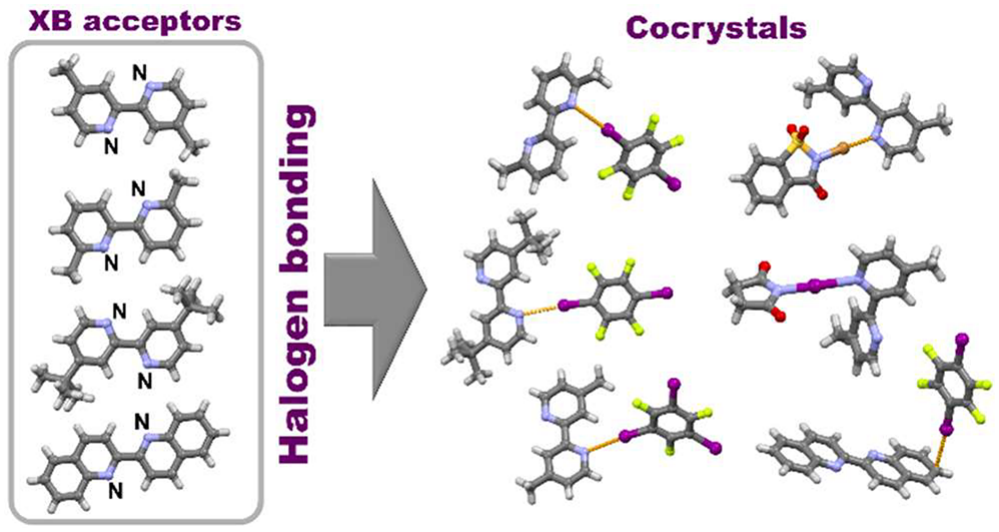

In this work, we present a systematic study of the halogen bonding potential of different 2,2′-bipyridine derivatives in the synthesis of cocrystals by using selected perfluorinated iodobenzenes and *N*-haloimides as halogen bond donors. These halogen bond acceptor molecules were chosen to explore how different substituents on 2,2′-bipyridine affect halogen bond formation. Out of 24 combinations, we obtained only 8 cocrystals by using two methods, liquid-assisted grinding and crystallization from the solution. Of those 8 cocrystals, one has already been described in the literature. As expected, structural data revealed that 2,2′-bipyridine derivatives act as ditopic halogen bond acceptors in all structures. Dominant interactions in 7 of the cocrystals are I···N or Br···N halogen bonds, while in the one remaining cocrystal it is the I···C(π) halogen bond.

## Abstract 2

In this work, we present a systematic study of the halogen bonding potential of different 2,2′-bipyridine derivatives in the synthesis of cocrystals with selected perfluorinated iodobenzenes and *N*-haloimides as halogen bond donors via liquid-assisted grinding and crystallization from the solution. Structural data revealed that 2,2′-bipyridine derivatives act as ditopic halogen bond acceptors in all structures.

The halogen bond (XB) is a noncovalent interaction between an electrophilic region on a covalently bonded halogen atom and a nucleophilic region, such as a lone electron pair of a Lewis base or delocalized π-electrons.^1,2^ Halogen bonds have become a very efficient tool in the crystal engineering toolkit for the synthesis of new functional materials,^3−5^ some of which have useful properties like photoresponsivity,^6,7^ mechanoresponsitivity,^8,9^ phosphorescence^10,11^ or magnetism.^12,13^ Halogen bonding as an interaction is closely related to hydrogen bonding, but there are several differences. First, halogen bond strength as well as hydrogen bond strength can be tuned by exchanging only one atom in the donor molecule,^14−17^ and furthermore, halogen bonds are more directional as a consequence of the specific localization of the electrophilic region, opposite the R–X covalent bond.^15,18^ Aside from changing the donor molecule, a commonly established route for tuning halogen bond strength is by changing the acceptor atom^16,19,20^ or by adding or changing substituents on the acceptor molecule.^17,21−25^ By using the substituent effect,^26^ it is possible to strengthen or weaken halogen bonding between molecules, regardless of whether the molecule is a halogen bond acceptor or donor. Previously, a diiodine basicity scale has been made, which quantifies the substituent effect on the XB basicity for a wide variety of Lewis bases.^27,28^ This scale shows that a small change in the molecular structure using very simple substituents can have an enormous effect on halogen bonding and its strength. For example, small- or medium-sized alkyl substituents such as methyl or butyl functional groups can increase diiodine basicity as a consequence of mainly the field/inductive effect, while groups that are bulkier such as isobutyl can decrease diiodine basicity as a result of mainly the steric effect. An overview of the currently available literature and Cambridge Structural Database (CSD)^29^ reveals that the most frequently recurring XB acceptor moieties are those containing oxygen or nitrogen atoms. There is a solid amount of data in the CSD for the [N, I–X] motif (26,579 data sets), X being any atom, and the [O, I–X] motif (19,608 data sets). Furthermore, in the [N, I–X] set, it was found that the N···I–X halogen bond was present in 2132 data sets (8.0%), while in the [O, I–X] set, the O···I–X halogen bond was present in 3248 data sets (16.6%). What is also worth mentioning is that in structures containing N···I–X halogen bonds, more than half of them have a pyridine nitrogen atom as an acceptor site (1095 data sets). When considering bipyridines as halogen bond acceptor moieties, 63 data sets were found with 4,4′-bipyridine, and only 25 data sets with 2,2′-bipyridine. In the vast majority of structures with bipyridines, perfluorinated halobenzenes were used as halogen bond donors. Therefore, we can clearly see that tertiary amines such as pyridine and its derivatives are found in most halogen-bonded adducts. Bipyridine derivatives as acceptor moieties are commonly used in crystal engineering because of their flexibility and ability to form different supramolecular architectures. 4,4′-bipyridine and its derivatives have been most extensively studied and are commonly used as ditopic and linear (rod-like) acceptor moieties.^30−32^ On the other hand, 2,2′-bipyridine and its derivatives have been poorly studied as halogen bond acceptors, although they are frequently used in analytical chemistry^33^ and as metal-chelating ligands due to their robust redox stability and ease of functionalization.^34^ 2,2′-bipyridine derivatives as halogen bond acceptors have been studied in terms of arylated derivatives with 1,4-diiodotetrafluorobenzene as a donor molecule,^35^ and in a recent study by Pennington and co-workers, 2,2′-bipyridine and 4,4′-dimethyl-2,2′-bipyridine have been cocrystallized with 1,4-dibromo- and 1,4-diiodotetrafluorobenzene as well as 4,4′-dibromo- and 4,4′-diiodooctafluorobiphenyl.^36^

In this study, we were interested in exploring how different substituents and their positions on 2,2′-bipyridine rings can affect halogen bond formation in cocrystals with selected halogen bond donors. For this purpose, we used four 2,2′-bipyridine derivatives: 4,4′-dimethyl-2,2′-bipyridine (**44diMebpy**), 4,4′-di-*tert*-butyl-2,2′-bipyridine (**44tBubpy)**, 6,6′-dimethyl-2,2′-bipyridine (**66diMebpy**), and 2,2′-biquinoline (**22biq**) (Scheme 1). As halogen bond donors, we selected both perfluorinated halobenzenes as well as *N*-haloimides (Scheme 1): 1,4-diiodotetrafluorobenzene (**14tfib**), 1,3,5-triiodotrifluorobenzene (**135tfib**), *N*-bromosuccinimide (**NBS**), *N*-iodosuccinimide (**NIS**), *N*-bromophthalimide (**NBF**) and *N*-bromosaccharine (**NBSac**).

**Scheme 1:**
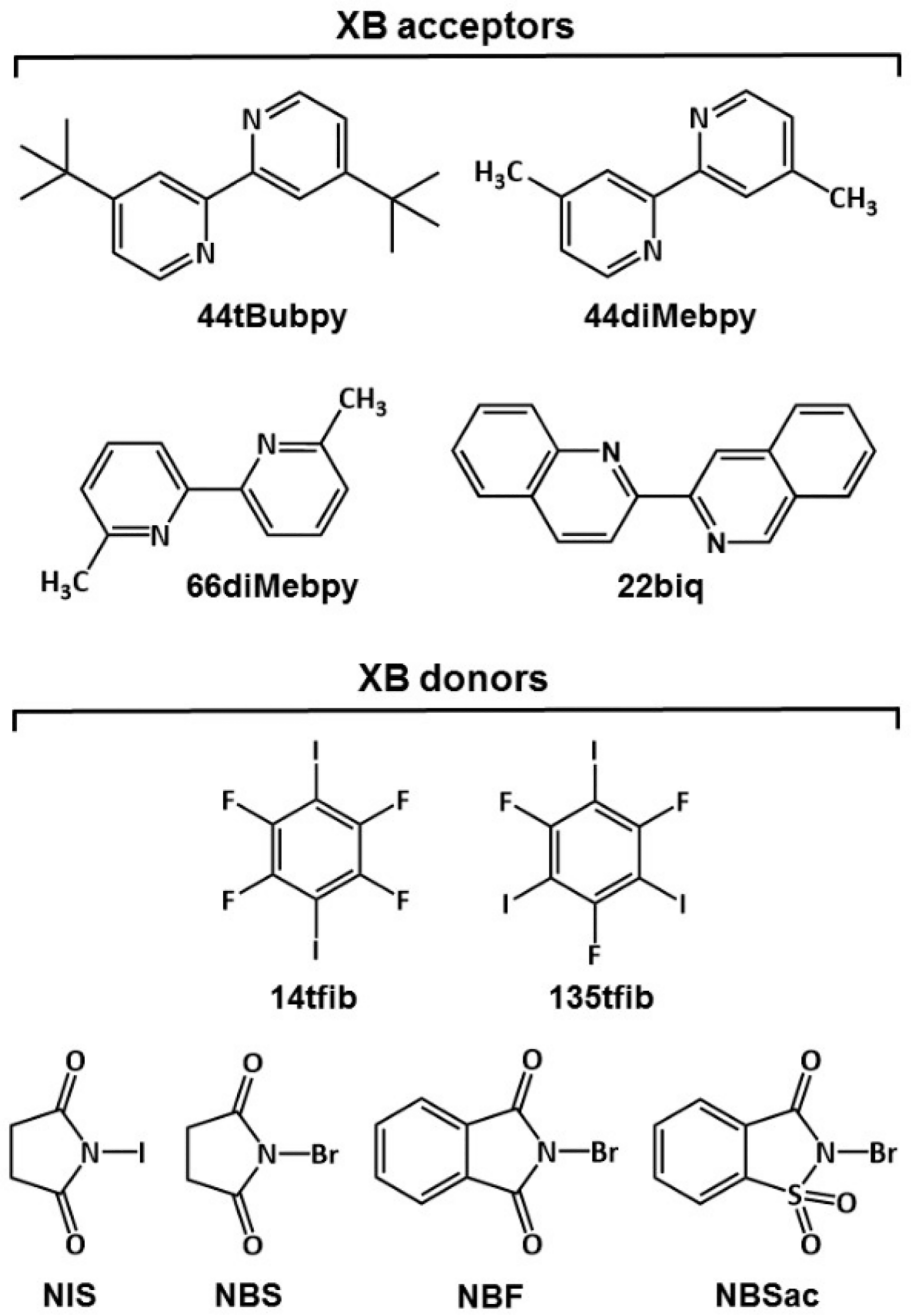
Structures of Halogen Bond (XB) Donor and Acceptor Molecules Used in This Study

Cocrystallization experiments were performed both mechanochemically, using liquid-assisted grinding (LAG),^37,38^ and from the solution. The mechanochemical reactions were performed in a Retsch MM200 mill using 10 mL stainless steel jars under ambient conditions (temperature ca. 25 °C, 40–60% relative humidity) for 15 min (see ESI). Reactants and products of the grinding experiments were characterized by powder X-ray diffraction (PXRD). In the interest of structural characterization of the obtained products, grinding experiments were followed by solution cocrystallization experiments. Single crystals were obtained by dissolving reactants in a solvent, after which the solutions were left to evaporate at room temperature. The obtained products were all characterized by single crystal X-ray diffraction (SCXRD) and differential scanning calorimetry (DSC). In order to rank 2,2′-bipyridine derivatives as halogen bond acceptors, values of molecular electrostatic potentials (MEPs) were calculated on geometries that were optimized using density functional theory (DFT). From Figure 1, it can be seen that nitrogen atoms **44tBubpy** and **44diMebpy** have the most negative electrostatic potential, with **44tBubpy** nitrogen atoms having the highest potential as acceptor sites (ΔMEP = 1.3 kJ mol^–1^*e*^–1^). A large decrease in the negativity of electrostatic potential on the nitrogen atom is observed in **66diMebpy** and especially in **22biq**, which can be expected to be a very poor halogen bond acceptor.

**Figure 1:**
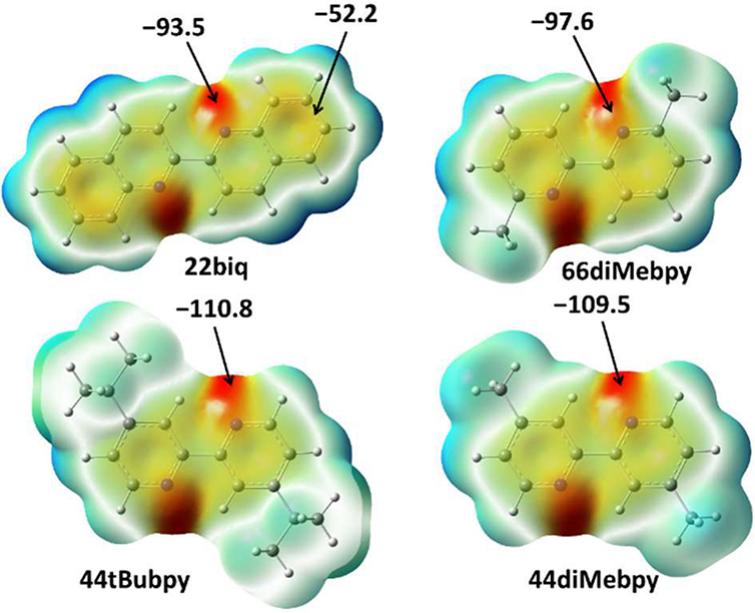
Calculated values in kJ mol^–1^*e*^–1^ of the molecular electrostatic potential mapped to the electron density isosurfaces (ρ = 0.001 au) corresponding to the optimized geometries of bipyridine derivatives (m062x/def2tzvp level of theory).

Screening experiments have shown that 8 cocrystals, out of a total of 24 reactant combinations, have been prepared both mechanochemically and by crystallization from the solution. Out of those 8 cocrystals, the 1:1 cocrystal of **44diMebpy** with **14tfib** has already been described in the literature by Pennington et al.^36^ Single crystal X-ray diffraction experiments on samples grown from solution have shown that the remaining seven newly obtained solids are cocrystals (Figures 2 and 3) of the following compositions: (**44diMebpy**)(**135tfib**)_2_, (**44diMebpy**)(**NIS**)_2_, (**44diMebpy**)(**NBSac**)_2_, (**66diMebpy**)_2_(**14tfib**), (**66diMebpy**)(**NIS**)_2_, (**44tBubpy**)(**14tfib**), and (**22biq**)(**14tfib**). The other 16 combinations resulted in either simple reactant mixtures, as in the case of unsuccessful combinations of acceptors and **135tfib** or **NBS**, or in unknown phases in combination with leftover reactants for **NBF** and **NBSac**, with possible donor decomposition through bromine evolution or bromination of the acceptor (see ESI, Tables S1 and S2 and Figures S8–S30). Dominant interactions in 6 of the obtained cocrystals are I···N or Br···N halogen bonds, while in the one remaining cocrystal it is the I···C(π) halogen bond. As expected, 2,2′-bipyridine derivatives act as ditopic halogen bond acceptors in all structures. Also, the obtained cocrystals could be classified based on the halogen bonding structural motifs mostly as either chains (1D) or discrete motifs (0D), while a layered structure (2D) is obtained only in one case.

**Figure 2:**
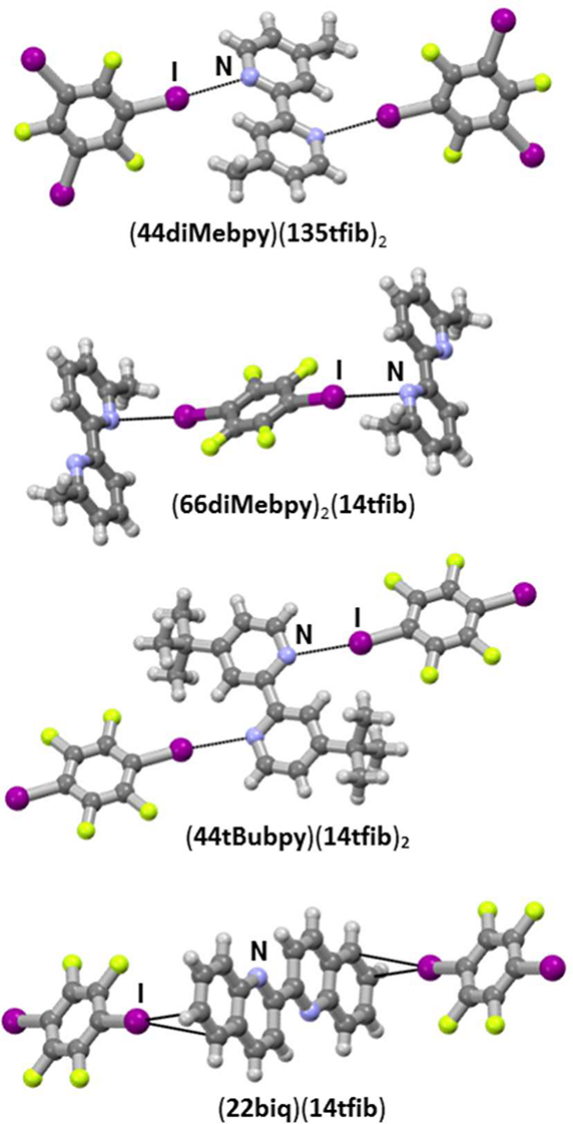
Parts of crystal structures of 2,2′-bipyridine derivative cocrystals with perfluorinated iodobenzenes.

**Figure 3:**
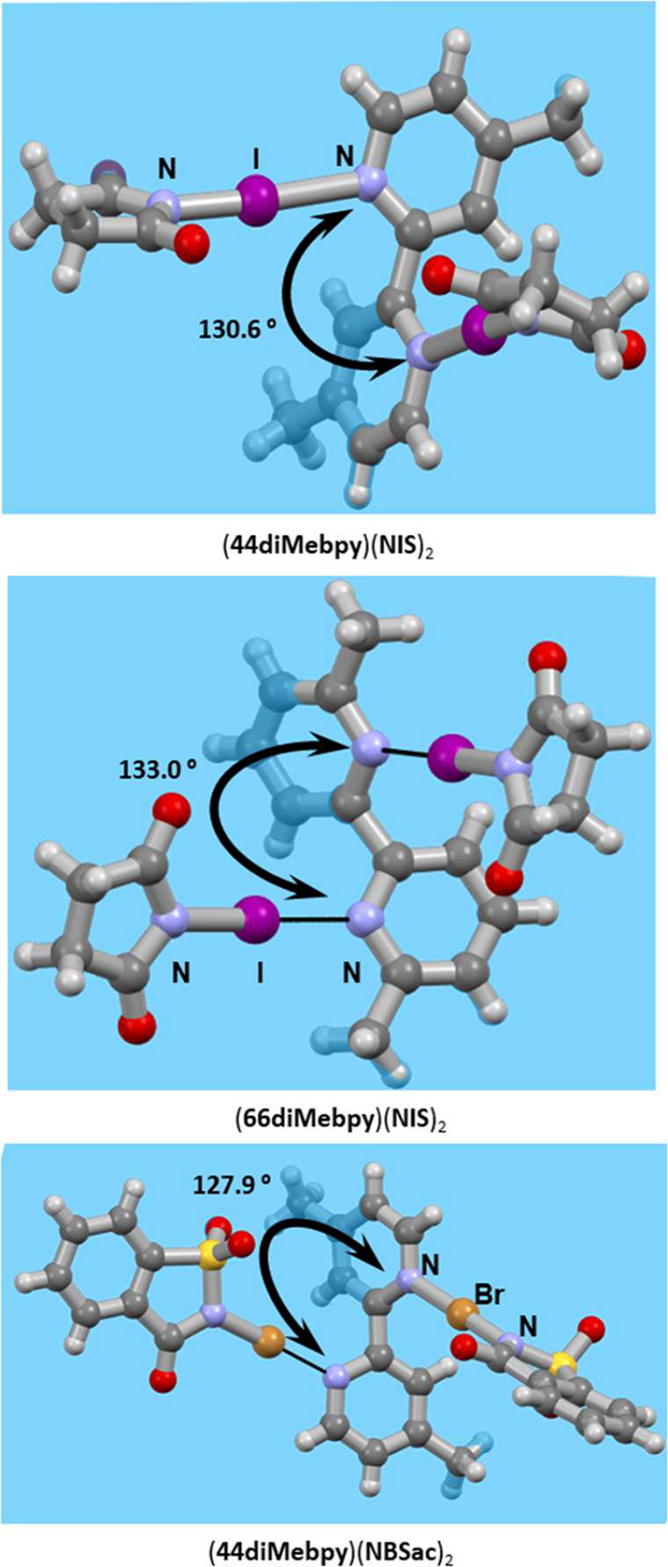
Parts of the crystal structures of cocrystals with *N*-haloimides as halogen bond donors. Bending of the bipyridine moiety is visualized using blue planes and torsional angle values.

Cocrystallization of **44diMebpy** gave cocrystals with **14tfib**, **135tfib**, **NIS**, and **NBSac**, although the **14tfib** cocrystal is structurally the same as that already characterized by Pennington et al.^36^ Structural characterization revealed that in the **14tfib** cocrystal,the structure consists of halogen-bonded chains of alternating donor and acceptor molecules, while in cocrystals with **NIS** and **NBSac**, there are discrete halogen-bonded complexes in which shorter halogen bonds were observed. In the **NBSac** cocrystal, there are two symmetrically inequivalent Br···N contacts (*d*(N1···Br1) = 2.202 Å, *d*(N2···Br2) = 2.356 Å), where the shortest contact has a pronouncedly high relative shortening (*RS*(N1···Br1) = 41.4%), while the **NIS** cocrystal features two symmetrically equivalent I···N contacts (*d*(I1···N1) = 2.515 Å). In (**44diMebpy**)(**135tfib**)_2_ only one iodine atom forms I···N halogen bonds (*d*(I1···N1) = 2.515 Å), another iodine atom participates in halogen bonding as both a donor and acceptor with two other **135tfib** molecules via I···I halogen bonds (*d*(I1···I2) = 3.831 Å), while the third iodine atom does not form any specific contacts with other molecules. Furthermore, the halogen bonded layers of (**44diMebpy**)(**135tfib**)_2_ are connected to other layers with C_methyl_–H···C(π) bonds (*d*(C6–H6C···C2) = 2.861 Å) between bipyridine molecules and C_methyl_–H···F contacts (*d*(C6–H6A···F3) = 3.246 Å) .

Two cocrystals were obtained with **66diMebpy** as a halogen bond acceptor, one with **14tfib** and another with **NIS**. In contrast to the 1:1 **44diMebpy** cocrystal with **14tfib**, the **66diMebpy** cocrystal with **14tfib** exhibits 2:1 acceptor to donor stoichiometry. In (**66diMebpy**)_2_(**14tfib**), there are two **66diMebpy** molecules independent by symmetry, only one of which participates in halogen bonding with **14tfib** through I···N contacts, therefore creating a chain of alternating donor and acceptor molecules. The second **66diMebpy** molecule is situated between two **14tfib** molecules of adjacent chains so that each chain is connected to the other one through π–π stacking interactions.

**44tBubpy** as a halogen bond acceptor gave only one cocrystal with **14tfib**. As in the previously mentioned structure of (**44diMebpy**)(**14tfib**), **44tBubpy** also forms chains of alternating donor and acceptor molecules, with one nitrogen on each side forming I···N halogen bonds (*d*(I1···N1) = 3.098 Å) with neighboring **14tfib** molecules. Although the nitrogen atom in **44tBubpy** could act as a better acceptor site based on electrostatic potential values than the one in **44diMebpy** (Figure 1), experimentally it was observed that **44diMebpy** forms halogen bonds much more readily, which will be discussed later.

In the only **22biq** cocrystal, (**22biq**)(**14tfib**), instead of the expected I···N halogen bond, I···C(π) bonds are formed (*d*(I1···C6) = 3.512 Å), leading to zigzag-shaped chains, where **22biq** acts as a ditopic halogen bond acceptor via the quinoline system benzene rings.

The only two 2,2′-bipyridines that exhibit halogen bonding with *N*-haloimides are **44diMebpy** and **66diMebpy**, cocrystals of which were obtained with **NIS** and **NBSac**. All cocrystals obtained with *N*-haloimides display significant bending of the acceptor’s bipyridine moiety. The torsional angle of 2,2′-bipyridine derivatives in those cocrystals is notably different from 180° (around 130°), whereas in cocrystals with perfluorinated halobenzenes, it is 180° or almost 180° (171.7° in (**44diMebpy**)(**14tfib**)). Additionally, it can be seen that there is correlation between relative shortening and a change in torsion angle, since greater relative shortening values result in greater deviations from the planarity of 2,2′-bipyridine (Figure 3). In the crystal structures of (**44diMebpy**)(**NIS**)_2_ and (**66diMebpy**)(**NIS**)_2_, each bipyridine molecule is involved in bonding with two **NIS** molecules via two identical I···N halogen bonds. The I···N distances are 2.112 Å (*RS* = 36.0%) for **44diMebpy** and 2.103 Å (*RS* = 33.3%) for **66diMebpy**, and are almost linear with N–I···N bond angles of 173.6° and 177.1°, respectively. Furthermore, the shortest halogen bonds were found in (**44diMebpy**)(**NBSac**)_2_ as stated above. Therefore, all of these observations indicate strong halogen bonds with partially covalent character. This behavior of *N-*haloimides when forming halogen bonds was previously reported by our group and by Rissanen and co-workers.^30,31,40,41^ Also, what can be observed from geometrical data presented in Table 1 is that symmetrical halogen bonds like N–I···N and N–Br···N, as is the case in cocrystals of *N*-haloimides, are shorter and more linear than the corresponding asymmetrical halogen bonds observed in cocrystals with perfluorinated halobenzenes.

**Table 1:** Halogen Bond Lengths *d*(X···A), Angles α, Relative Shortening *RS*^b^ of X···A Distances, and Torsional Angles β_tor._ for Bipyridine Derivatives in the Herein Prepared Cocrystals

Cocrystal	D–X···A	*d*(D–X) / Å	*d*(X···A) / Å	α(D–X···A) / °	*RS*^b^ / %	β_tor._ / °
(**44diMebpy**)(**14tfib**)^a^	C13–I1···N1	2.095	3.128	170.9	20.4	171.7
(**44diMebpy**)(**135tfib**)_2_	C7–I1···N1	2.092	3.054	173.1	22.3	180
	C9–I2···I2		3.831	176.0	3.3	—
(**44diMebpy**)(**NIS**)_2_	N1–I1···N2	2.112	2.515	173.6	36.0	130.6
(**44diMebpy**)(**NBSac**)_2_	N4–Br2···N2	1.928	2.356	178.0	37.3	127.9
	N3–Br1···N1	2.010	2.202	178.9	41.4	
(**66diMebpy**)_2_(**14tfib**)	C13–I1···N1	2.071	3.277	171.3	16.6	180
(**66diMebpy**)(**NIS**)_2_	N1–I1···N2	2.103	2.620	177.1	33.3	133.0
(**44tBubpy**)(**14tfib**)	C10–I1···N1	2.081	3.098	173.8	21.7	180
(**22biq**)(**14tfib**)	C10–I1···C6	2.075	3.512	173.8	13.1	180

a Cocrystal that was already prepared by Pennington and co-workers.^36^

b *RS* = 1 – *d*(D···A)/[*r*_vdW_(D) + *r*_vdW_(A)].^39^

In general, alkyl groups such as methyl and *tert*-butyl are poor electron-donating groups and can affect the ability of the nitrogen atom as an acceptor site. In comparison with the diiodine p*K*_BI2_ scale derived by Laurence et al.^28^ for methyl and *tert*-butyl pyridine derivatives, it can be seen that the acceptor potential of nitrogen rises by the distancing of the methyl group from the nitrogen atom. Also, in the case of 4-methylpyridine and 4-*tert*-butylpyridine, it can be seen that the *tert*-butyl derivative has a higher p*K*_BI2_ value, meaning higher nitrogen atom acceptor ability. From data obtained by our experiments, it is clear that for methylated 2,2′-bipyridine derivatives, there is correlation between calculated MEPs, crystallographic data, and the diiodine basicity scale. Although there is evidence both from the diiodine basicity scale and MEPs that **44tBubpy** should be the best acceptor, our experimental data point out that this is not the case. This observation can be explained by invoking steric effects, mainly the presence of the second pyridine ring, which also contains the *tert*-butyl group. Because of the *trans*- conformation of the bipyridine system, the nitrogen atom is hindered by *tert*-butyl groups, which afterward decreases the ability for halogen bond formation, especially with bulkier halogen bond donors.

Thermal analysis by DSC in combination with PXRD revealed that the obtained cocrystals are pure phases. DSC curves for cocrystals with perfluorinated halobenzenes exhibit one well-defined endothermic peak that corresponds to melting. The only exception is (**66diMebpy**)_2_(**14tfib**), where the DSC curve features two endothermic peaks in the range between 60 and 80 °C. Melting points of most cocrystals with perfluorinated iodobenzenes fall in the range between 100 and 160 °C, while the (**66diMebpy**)_2_(**14tfib**) cocrystal has a melting point below 100 °C (Table 2). Thermal analysis of *N*-haloimide cocrystals shows that the DSC curve for (**66diMebpy**)(**NIS**)_2_ features one small endothermic peak that corresponds to melting, followed by a sharp exothermic peak, while all other *N*-haloimide cocrystals feature only the exothermic peak that can be attributed to explosive decomposition. These exothermic peaks are in the range from 130 to 155 °C.

**Table 2:** Melting Points and Exothermic Peaks of the Obtained Cocrystals Determined by DSC Experiments

Cocrystal	Melting point / °C	Exothermic peak / °C
(**66diMebpy**)_2_(**14tfib**)	70.2	—
(**44tBubpy**)(**14tfib**)	142.8	—
(**22biq**)(**14tfib**)	159.6	—
(**44diMebpy**)(**135tfib**)_2_	109.9	—
(**44diMebpy**)(**NIS**)_2_	—	148.1
(**66diMebpy**)(**NIS**)_2_	144.9	152.7
(**44diMebpy**)(**NBSac**)_2_	—	132.2

To conclude, in this work, we have systematically explored the effect of different substituents on the pyridine nitrogen atom to act as a halogen bond acceptor for cocrystal formation with selected 2,2′-bipyridines and perfluorinated halobenzenes and *N*-haloimides as halogen bond donors. The herein prepared seven cocrystals with the previously reported (**44diMebpy**)(**14tfib**),^36^ show that **44diMebpy** has the highest ability for halogen bond formation. Also, nitrogen atoms in **22biq** have the lowest ability for the formation of halogen bonds, which is confirmed both experimentally and by theoretical considerations. The nitrogen atom in **22biq** does not participate in halogen bonding; instead, the benzene ring acts as the acceptor site. Halogen bonds with highly covalent character were reported in cocrystals with *N*-haloimides, and bending of the bipyridine system also occurred. In all structures, 2,2′-bipyridine derivatives acted as ditopic acceptors in a *trans-* conformation. Both DFT calculations and p*K*_BI2_ scale values are consistent with our experimental data for methylated derivatives, meaning that an increase in the nitrogen atom acceptor potential is associated with an increased distance of the substituent from the nitrogen atom. Interestingly, although DFT calculations and the previously derived diiodine basicity scale show that the most negative electrostatic potential is on the **44tBubpy** nitrogen atom, only one cocrystal was obtained with this acceptor, which can be attributed to large *tert*-butyl groups in the close vicinity of nitrogen atoms that by steric effects prevent or hinder halogen bond formation. These results present how different substituents and their positions can be used to modify the acceptor potential of an atom and therefore be used in the synthesis of new materials based on halogen bonding.

## Supporting Information Available

Experimental details, including syntheses, single crystal diffraction data, PXRD patterns and DSC curves (DOCX)
