# Supplementary material for: 2,2′-Bipyridine Derivatives as Halogen Bond Acceptors in Multicomponent Crystals
Source: Cryst Growth Des. 2023 Nov 9;23(12):8482–7. doi: 10.1021/acs.cgd.3c01055 (PMC10711937; doi:10.1021/acs.cgd.3c01055)
Supplement: Supplementary file 1 — cg3c01055_si_001.docx [file cg3c01055_si_001.docx]

**SUPPORTING INFORMATION**

**2,2'-bipyridine Derivatives as Halogen Bond Acceptors in Multicomponent Crystals**

Filip Kučas, Lidija Posavec, Vinko Nemec, Nikola Bedeković and Dominik Cinčić*

Department of Chemistry, Faculty of Science, University of Zagreb, Horvatovac 102a,
HR-10000 Zagreb, Croatia

Email: dominik@chem.pmf.hr

Fax: +385 1 4606 341

Tel: +385 1 4606 362

**Table of Contents**

| Experimental details | Mechanochemical experiments (Table S1.), solution experiments (Table S2.), thermal analysis, powder X-ray diffraction experiments, single crystal X-ray diffraction experiments, computational details | 4 |
| --- | --- | --- |
|  |  |  |
| Table S3. | Crystal data and refinement details for the prepared cocrystals. | 9 |
|  |  |  |
| Figure S1. | Molecular structure of (44diMebpy)(135tfib)_2_ showing the atom-labeling scheme. Displacement ellipsoids are drawn at the 50 % probability level, and H atoms are shown as small spheres of arbitrary radius. | 13 |
|  |  |  |
| Figure S2. | Molecular structure of (44diMebpy)(NIS)_2_ showing the atom-labeling scheme. Displacement ellipsoids are drawn at the 50 % probability level, and H atoms are shown as small spheres of arbitrary radius. | 13 |
|  |  |  |
| Figure S3. | Molecular structure of (44diMebpy)(NBSac)_2_ showing the atom-labeling scheme. Displacement ellipsoids are drawn at the 50 % probability level, and H atoms are shown as small spheres of arbitrary radius. | 14 |
|  |  |  |
| Figure S4. | Molecular structure of (66diMebpy)_2_(14tfib) showing the atom-labeling scheme. Displacement ellipsoids are drawn at the 50 % probability level, and H atoms are shown as small spheres of arbitrary radius. | 14 |
|  |  |  |
| Figure S5. | Molecular structure of (66diMebpy)(NIS)_2_ showing the atom-labeling scheme. Displacement ellipsoids are drawn at the 50 % probability level, and H atoms are shown as small spheres of arbitrary radius. | 15 |
|  |  |  |
| Figure S6. | Molecular structure of (44tBubpy)(14tfib) showing the atom-labeling scheme. Displacement ellipsoids are drawn at the 50 % probability level, and H atoms are shown as small spheres of arbitrary radius. | 15 |
|  |  |  |
| Figure S7. | Molecular structure of (22biq)(14tfib) showing the atom-labeling scheme. Displacement ellipsoids are drawn at the 50 % probability level, and H atoms are shown as small spheres of arbitrary radius. | 16 |
|  |  |  |
| Figure S8. | PXRD patterns of: a) 44diMebpy, b) 135tfib, c) product obtained by grinding 44diMebpy and 135tfib in a 1:1 stoichiometric ratio d) calculated pattern from single crystal data. | 17 |
|  |  |  |
| Figure S9. | PXRD patterns of: a) 44diMebpy, b) NIS, c) product obtained by grinding 44diMebpy and NIS in a 1:2 stoichiometric ratio d) calculated pattern from single crystal data. | 17 |
|  |  |  |
| Figure S10. | PXRD patterns of: a) 44diMebpy, b) NBF, c) product obtained by grinding 44diMebpy and NBF in a 1:1 stoichiometric ratio for 10 minutes, d) product obtained by grinding 44diMebpy and NBF in a 1:1 stoichiometric ratio for 30 minutes, e) bulk product obtained by dissolving 44diMebpy and NBF in a 1:1 stoichiometric ratio in tetrahydrofuran, f) bulk product obtained by dissolving 44diMebpy and NBF in a 1:1 stoichiometric ratio in acetonitrile. | 18 |
|  |  |  |
| Figure S11. | PXRD patterns of: a) 44diMebpy, b) NBSac, c) product obtained by grinding 44diMebpy and NBSac in a 1:2 stoichiometric ratio d) calculated pattern from single crystal data. | 18 |
|  |  |  |
| Figure S12. | PXRD patterns of: a) 44diMebpy, b) NBS, c) product obtained by grinding 44diMebpy and NBS in a 1:1 stoichiometric ratio. | 19 |
|  |  |  |
| Figure S13. | PXRD patterns of: a) 66diMebpy, b) 14tfib, c) product obtained by grinding 66diMebpy and 14tfib in a 2:1 stoichiometric ratio d) calculated pattern from single crystal data. | 19 |
|  |  |  |
| Figure S14. | PXRD patterns of: a) 66diMebpy calculated from single crystal data, b) 135tfib calculated from single crystal data, c) product obtained by grinding 66diMebpy and 135tfib in a 2:1 stoichiometric ratio. | 20 |
|  |  |  |
| Figure S15. | PXRD patterns of: a) 66diMebpy, b) NIS, c) product obtained by grinding 66diMebpy and NIS in a 1:2 stoichiometric ratio d) calculated pattern from single crystal data. | 20 |
|  |  |  |
| Figure S16. | PXRD patterns of: a) 66diMebpy, b) NBF, c) product obtained by grinding 66diMebpy and NBF in a 1:1 stoichiometric ratio for 10 minutes, d) product obtained by grinding 66diMebpy and NBF in a 1:1 stoichiometric ratio for 30 minutes, e) bulk product obtained by dissolving 66diMebpy and NBF in a 1:1 stoichiometric ratio in acetonitrile. | 21 |
|  |  |  |
| Figure S17. | PXRD patterns of: a) 66diMebpy, b) NBSac, c) product obtained by grinding 66diMebpy and NBSac in a 1:1 stoichiometric ratio, d) bulk product obtained by dissolving 66diMebpy and NBSac in a 1:1 stoichiometric ratio in dichloromethane. | 21 |
|  |  |  |
| Figure S18. | PXRD patterns of: a) 66diMebpy, b) NBS, c) product obtained by grinding 66diMebpy and NBS in a 1:1 stoichiometric ratio. | 22 |
|  |  |  |
| Figure S19. | PXRD patterns of: a) 44tBubpy, b) 14tfib, c) product obtained by grinding 44tBubpy and 14tfib in a 1:1 stoichiometric ratio d) calculated pattern from single crystal data. | 22 |
|  |  |  |
| Figure S20. | PXRD patterns of: a) 44tBubpy calculated from single crystal data, b) 135tfib calculated from single crystal data, c) product obtained by grinding 44tBubpy and 135tfib in a 1:1 stoichiometric ratio. | 23 |
|  |  |  |
| Figure S21. | PXRD patterns of: a) 44tBubpy calculated from single crystal data, refcode MUBJOJ, b) NIS, c) NHS calculated from single crystal data, refcode SUCCIN, d) product obtained by grinding 44tBubpy and NIS in a 1:1 stoichiometric ratio, e) product obtained by dissolving 44tBubpy and NIS in a 1:1 stoichiometric ratio in tetrahydrofuran. | 23 |
|  |  |  |
| Figure S22. | PXRD patterns of: a) 44tBubpy calculated from single crystal data, refcode MUBJOJ, b) NBF, c) product obtained by grinding 44tBubpy and NBF in a 1:1 stoichiometric ratio for 10 minutes, d) product obtained by grinding 44tBubpy and NBF in a 1:1 stoichiometric ratio for 30 minutes, e) product obtained by dissolving 44tBubpy and NBF in a 1:1 stoichiometric ratio in tetrahydrofuran, f) product obtained by dissolving 44tBubpy and NBF in a 1:1 stoichiometric ratio in acetonitrile. | 24 |
|  |  |  |
| Figure S23. | PXRD patterns of: a) 44tBubpy calculated from single crystal data, refcode MUBJOJ, b) NBSac, c) product obtained by grinding 44tBubpy and NBSac in a 1:1 stoichiometric ratio for 10 minutes, d) product obtained by dissolving 44tBubpy and NBSac in a 1:1 stoichiometric ratio in dichloromethane. | 24 |
|  |  |  |
| Figure S24. | PXRD patterns of: a) 44tBubpy calculated from single crystal data, refcode MUBJOJ, b) 44tBubpy, reactant powder pattern, c) NBS, d) product obtained by grinding 44tBubpy and NBS in a 1:1 stoichiometric ratio. | 25 |
|  |  |  |
| Figure S25. | PXRD patterns of: a) 22biq, b) 14tfib, c) product obtained by grinding 22biq and 14tfib in a 1:1 stoichiometric ratio d) calculated pattern from single crystal data. | 25 |
|  |  |  |
| Figure S26. | PXRD patterns of: a) 22biq calculated from single crystal data, b) 135tfib calculated from single crystal data, c) product obtained by grinding 22biq and 135tfib in a 1:1 stoichiometric ratio, d) product obtained by grinding 22biq and 135tfib in a 2:1 stoichiometric ratio. | 26 |
|  |  |  |
| Figure S27. | PXRD patterns of: a) 22biq calculated from single crystal data, b) NIS, c) product obtained by grinding 22biq and NIS in a 1:1 stoichiometric ratio. | 26 |
|  |  |  |
| Figure S28. | PXRD patterns of: a) 22biq calculated from single crystal data, b) NBF, c) product obtained by grinding 22biq and NBF for 10 minutes in a 1:1 stoichiometric ratio, d) product obtained by grinding 22biq and NBF for 30 minutes in a 1:1 stoichiometric ratio, e) crystallization bulk obtained by dissolving 22biq and NBF in acetonitrile in a 1:1 stoichiometric ratio and letting the solvent evaporate at room temperature. | 27 |
|  |  |  |
| Figure S29. | PXRD patterns of: a) 22biq calculated from single crystal data, b) NBSac, c) product obtained by grinding 22biq and NBSac in a 1:1 stoichiometric ratio. | 27 |
|  |  |  |
| Figure S30. | PXRD patterns of: a) 22biq calculated from single crystal data, b) NBS, c) product obtained by grinding 22biq and NBS in a 1:1 stoichiometric ratio. | 28 |
|  |  |  |
| Figure S31. | DSC curve of (44diMebpy)(135tfib)_2_. | 29 |
|  |  |  |
| Figure S32. | DSC curve of (44diMebpy)(NIS)_2_. | 29 |
|  |  |  |
| Figure S33. | DSC curve of (44diMebpy)(NBSac)_2_. | 30 |
|  |  |  |
| Figure S34. | DSC curve of (66diMebpy)_2_(14tfib). | 30 |
|  |  |  |
| Figure S35. | DSC curve of (66diMebpy)(NIS)_2_. | 31 |
|  |  |  |
| Figure S36. | DSC curve of (44tBubpy)(14tfib). | 31 |
|  |  |  |
| Figure S37. | DSC curve of (22biq)(14tfib). | 32 |
|  |  |  |
|  |  |  |

**Experimental details**

**Mechanochemical experiments**

**Table S1.** Experimental data for mechanochemical experiments. Reaction mixtures were placed in 10 mL stainless steel jars along with 10 μL of acetonitrile and one stainless steel ball 7 mm in diameter. The reaction mixtures were then milled in a Retsch MM200 Shaker Mill operating at 25 Hz

| acceptor | *m* / mg | donor | *m* / mg | *t* / min | result |
| --- | --- | --- | --- | --- | --- |
| 44diMebpy | 15.9 | **135tfib** | 44.1 | 20 | (**44diMebpy**)(**135tfib**)_2_ |
| 44diMebpy | 17.4 | **NIS** | 42.6 | 10 | (**44diMebpy**)(**NIS**)_2_ |
| 44diMebpy | 27.1 | **NBF** | 33.3 | 10 | unknown phase + **NBF** |
| 44diMebpy | 26.9 | **NBF** | 33.1 | 30 | unknown phase + **NBF** |
| 44diMebpy | 15.6 | **NBSac** | 44.4 | 10 | (**44diMebpy**)(**NBSac**)_2_ |
| 44diMebpy | 30.6 | **NBS** | 29.6 | 10 | reactant mixture |
| 44tBubpy | 24.0 | **14tfib** | 36.0 | 10 | (**44tBubpy**)(**14tfib**) |
| 44tBubpy | 21.1 | **135tfib** | 39.3 | 20 | reactant mixture |
| 44tBubpy | 32.6 | **NIS** | 27.6 | 20 | unknown phase + **44tBubpy** |
| 44tBubpy | 32.5 | **NBF** | 27.5 | 10 | unknown phase + reactant mixture |
| 44tBubpy | 33.5 | **NBF** | 27.8 | 30 | unknown phase + reactant mixture |
| 44tBubpy | 30.8 | **NBSac** | 29.9 | 10 | mostly amorphous, unknown phase + **44tBubpy** |
| 44tBubpy | 36.3 | **NBS** | 24.4 | 10 | reactant mixture |
| 66diMebpy | 28.7 | **14tfib** | 31.3 | 20 | (**66diMebpy**)_2_(**14tfib**) |
| 66diMebpy | 25.6 | **135tfib** | 35.1 | 20 | reactant mixture |
| 66diMebpy | 17.4 | **NIS** | 42.6 | 10 | (**66diMebpy**)(**NIS**)_2_ |
| 66diMebpy | 27.0 | **NBF** | 32.9 | 10 | unknown phase + reactant mixture |
| 66diMebpy | 27.0 | **NBF** | 32.9 | 30 | unknown phase + reactant mixture |
| 66diMebpy | 25.2 | **NBSac** | 35.8 | 10 | poorly crystalline; unknown phase + **NBSac** + **66diMebpy** |
| 66diMebpy | 30.7 | **NBS** | 29.5 | 10 | reactant mixture |
| 22biq | 23.4 | **14tfib** | 36.6 | 20 | (**22biq**)(**14tfib**) |
| 22biq | 21.1 | **135tfib** | 40.0 | 20 | reactant mixture |
| 22biq | 30.0 | **135tfib** | 30.0 | 20 | reactant mixture |
| 22biq | 31.8 | **NIS** | 28.7 | 20 | reactant mixture |

**Table S1.** continued

| acceptor | *m* / mg | donor | *m* / mg | *t* / min | result |
| --- | --- | --- | --- | --- | --- |
| 22biq | 32.0 | **NBF** | 28.2 | 10 | unknown phase + reactant mixture |
| 22biq | 31.9 | **NBF** | 28.0 | 30 | unknown phase + reactant mixture |
| 22biq | 29.7 | **NBSac** | 30.9 | 10 | poorly crystalline; reactant mixture |
| 22biq | 35.4 | **NBS** | 24.7 | 10 | reactant mixture |

**Solution experiments**

**Table S2.** Experimental data for solution experiments. Reaction mixtures were carefully heated to dissolve the reactants, if necessary, and then left to crystallize at room temperature.

| reaction mixture | stoichiometric ratio | solvent | result |
| --- | --- | --- | --- |
| 15.9 mg 44diMebpy  44.1 mg 135tfib | 1:1 | 2.0 mL dichloromethane | (**44diMebpy**)(**135tfib**)_2_ single crystals |
| 17.4 mg 44diMebpy 42.6 mg NIS | 1:2 | 1.0 mL dichloromethane + 1.0 mL acetone | (**44diMebpy**)(**NIS**)_2_ single crystals |
| 27.1 mg 44diMebpy 33.3 mg NBF | 1:1 | 2.0 mL tetrahydrofuran | unknown phase + NBF |
| 27.1 mg 44diMebpy 33.3 mg NBF | 1:1 | 2.0 mL acetonitrile | **44diMebpy** + amorphous bulk |
| 15.6 mg 44diMebpy 44.4 mg NBSac | 1:2 | 1.0 mL dichloromethane + 1.0 mL acetone | (**44diMebpy**)(**NBSac**)_2_ single crystals |
| 24.0 mg 44tBubpy 36.0 mg 14tfib | 1:1 | 1.0 mL dichloromethane + 1.0 mL ethanol | (**44tBubpy**)(**14tfib**) single crystals |
| 32.6 mg 44tBubpy 27.6 mg NIS | 1:1 | 2.0 mL tetrahydrofuran | unknown phase + mixture of **NHS** and **44tBubpy** |
| 32.5 mg 44tBubpy 27.5 mg NBF | 1:1 | 2.0 mL tetrahydrofuran | reactant mixture |
| 32.5 mg 44tBubpy 27.5 mg NBF | 1:1 | 2.0 mL acetonitrile | reactant mixture (predominately crystalline **44tBubpy**) |
| 28.7 mg 66diMebpy 31.3 mg 14tfib | 2:1 | 2.0 ml methanol | (**66diMebpy**)_2_(**14tfib**) single crystals |
| 17.4 mg 66diMebpy 42.6 mg NIS | 1:2 | 1.0 mL dichloromethane + 1.0 mL acetone | (**66diMebpy**)(**NIS**)_2_ single crystals |
| 27.0 mg 66diMebpy 33.0 mg NBF | 1:1 | 2.0 mL tetrahydrofuran | oil/glassy residue |

**Table S2.** continued

| reaction mixture | stoichiometric ratio | solvent | result |
| --- | --- | --- | --- |
| 27.0 mg 66diMebpy 33.0 mg NBF | 1:1 | 2.0 mL acetonitrile | unknown phase + reactant mixture |
| 25.2 mg 66diMebpy 35.8 mg NBSac | 1:1 | 1.0 mL dichloromethane + 1.0 mL acetone | unknown phase + **NBSac** + **NHSac** + **66diMebpy** |
| 25.2 mg 66diMebpy 35.8 mg NBSac | 1:1 | 1.0 mL dichloromethane + 1.0 mL ethanol | bromine evolution/ bromination in solution |
| 23.4 mg 22biq 36.6 mg 14tfib | 1:1 | 2.0 mL chloroform | (**22biq**)(**14tfib**)  single crystals |
| 21.1 mg 22biq 40.0 mg 135tfib | 1:1 | 2.0 mL chloroform | oil/glassy residue |
| 21.1 mg 22biq 40.0 mg 135tfib | 1:1 | 1.0 mL chloroform  + 1.0 mL ethanol | oil/glassy residue |
| 21.1 mg 22biq 40.0 mg 135tfib | 1:1 | 2.0 mL dichloromethane | oil/glassy residue |
| 21.1 mg 22biq 40.0 mg 135tfib | 1:1 | 2.0 mL dichloromethane + 1.0 mL ethanol | oil/glassy residue |
| 21.1 mg 22biq 40.0 mg 135tfib | 1:1 | 1.0 mL chloroform + 1.0 mL dichloromethane | oil/glassy residue |
| 32.0 mg 22biq 28.2 mg NBF | 1:1 | 2.0 mL tetrahydrofuran | oil/glassy residue |
| 32.0 mg 22biq 28.2 mg NBF | 1:1 | 2.0 mL acetonitrile | **22biq** |

**Thermal analysis**

DSC measurements were performed on a Mettler-Toledo DSC823^e^ module. The samples were placed in sealed aluminium pans (40 μL) with two pinholes made on the top cover, and heated in flowing nitrogen (50 mL min^−1^) from 25 °C to 500 °C at a rate of 10 °C min^−1^. The data collection and analysis was performed using the program package STAR^e^ Software 15.00.^1^

**Powder X-ray diffraction experiments**

PXRD experiments were performed on a Malvern PANalytical X-ray diffractometer with Cu*K*α1 (1.54056 Å) radiation at 15 mA and 40 kV. The scattered intensities were measured with a scintillation counter. The angular range was from 5 to 40° (2*θ* ) with steps of 0.02 – 0.03°, and the measuring time was 0.2 – 0.5 s per step. Data collection and analysis was performed using the program package Data Viewer.^2^

**Single-crystal X-ray diffraction experiments**

The crystal and molecular structures of the prepared cocrystals were determined by single crystal X-ray diffraction. Details of data collection and crystal structure refinement are listed in Table S1, S2, S3 and S4. Diffraction measurements were made on an Oxford Diffraction Xcalibur Kappa CCD X-ray diffractometer and Rigaku Synergy XtaLAB X-ray diffractometer with graphite-monochromated MoKα (*λ* = 0.71073Å) radiation. The data sets were collected using the ω scan mode over the 2*θ* range up to 54° (Xcalibur Kappa CCD) and up to 64° (Synergy XtaLAB). Programs CrysAlis CCD, CrysAlis RED and CrysAlisPro were employed for data collection, cell refinement, and data reduction.^3,4^ The structures were solved by direct methods and refined using the SHELXS, SHELXT, and SHELXL programs, respectively.^5,6^ The structural refinement was performed on *F*^2^ using all data. Hydrogen atoms were placed in calculated positions and treated as riding on their parent atoms. All calculations were performed using the WINGX crystallographic suite of programs.^7^ The molecular structures of compounds and their molecular packing projections were prepared by Mercury.^8^

**Computational Details.** All calculations were performed using Gaussian 16 software package.^9^ Geometry optimizations were performed using M062X/def2-tzvp level of theory,^10^ with ultrafine integration grid (99 radial shells and 590 points per shell). The default Gaussian convergence criteria were used. Harmonic frequency calculations were performed on the optimized geometries to ensure the success of each geometry optimization. The Figures were prepared using GaussView.^11^

**References**

1. STARe Evaluation Software Version 15.00, Mettler–Toledo GmbH, 2016.

2. Data Viewer Version 1.9a, PANalytical B.V. Amelo, The Netherlands, 2018.

3. Oxford Diffraction, Oxford Diffraction Ltd., Xcalibur CCD system, CrysAlis CCD and CrysAlis RED software, Version 1.170, 2003.

4. Rigaku Oxford Diffraction, Gemini CCD system, CrysAlis Pro software, Version 171.41.93a, 2020.

5. (a) G. M. Sheldrick, *Acta Cryst. A*, 2008, **64**, 112–122; (b) G. M. Sheldrick, *Acta Cryst. C*, 2015, **71**, 3–8.

6. G. M. Sheldrick, *Acta Cryst. A*, 2015, **71**, 3–8.

7. L. J. Farrugia, *J. Appl. Cryst.*, 2012, **45**, 849–854.

8. C. F. Macrae, I. J. Bruno, J. A. Chisholm, P. R. Edgington, P. McCabe, E. Pidcock, L. Rodriguez-Monge, R. Taylor, J. v. d. Streek and P. A. Wood, *J. Appl. Crystallogr.* **2008**, 41, 466.

9. Gaussian 16, Revision C.01, Frisch, M. J.; Trucks, G. W.; Schlegel, H. B.; Scuseria, G. E.; Robb, M. A.; Cheeseman, J. R.; Scalmani, G.; Barone, V.; Petersson, G. A.; Nakatsuji, H.; Li, X.; Caricato, M.; Marenich, A. V.; Bloino, J.; Janesko, B. G.; Gomperts, R.; Mennucci, B.; Hratchian, H. P.; Ortiz, J. V.; Izmaylov, A. F.; Sonnenberg, J. L.; Williams-Young, D.; Ding, F.; Lipparini, F.; Egidi, F.; Goings, J.; Peng, B.; Petrone, A.; Henderson, T.; Ranasinghe, D.; Zakrzewski, V. G.; Gao, J.; Rega, N.; Zheng, G.; Liang, W.; Hada, M.; Ehara, M.; Toyota, K.; Fukuda, R.; Hasegawa, J.; Ishida, M.; Nakajima, T.; Honda, Y.; Kitao, O.; Nakai, H.; Vreven, T.; Throssell, K.; Montgomery, J. A., Jr.; Peralta, J. E.; Ogliaro, F.; Bearpark, M. J.; Heyd, J. J.; Brothers, E. N.; Kudin, K. N.; Staroverov, V. N.; Keith, T. A.; Kobayashi, R.; Normand, J.; Raghavachari, K.; Rendell, A. P.; Burant, J. C.; Iyengar, S. S.; Tomasi, J.; Cossi, M.; Millam, J. M.; Klene, M.; Adamo, C.; Cammi, R.; Ochterski, J. W.; Martin, R. L.; Morokuma, K.; Farkas, O.; Foresman, J. B.; Fox, D. J. Gaussian, Inc., Wallingford CT, 2016.

10. Zhao, Y.; Truhlar, D.G. The M06 suite of density functionals for main group thermochemistry, thermochemical kinetics, noncovalent interactions, excited states, and transition elements: Two new functionals and systematic testing of four M06-class functionals and 12 other functionals. *Theor. Chem. Acc.*, 2008, **120**, 215–241.

11. GaussView, Version 5.1; Dennington, R.; Keith, T.A.; Millam, J.M. (Eds.) Semichem Inc.: Shawnee, KS, USA, 2008.

**Table S3.** Crystal data and refinement details for the prepared compounds.

|  | (**44diMebpy**)(**135tfib**)_2_ | (**44diMebpy**)(**NIS**)_2_ |
| --- | --- | --- |
| Molecular formula | (C_12_H_12_N_2_)(C_6_F_3_I_3_)_2_ | (C_12_H_12_N_2_)(C_4_H_4_INO_2_)_2_ |
| *M*_r_ | 1203.76 | 634.20 |
| Crystal system | monoclinic | orthorombic |
| Space group | *P* 2_1_/*n* | *P* *bcn* |
| Crystal data: |  |  |
| *a* / Å | 8.6021(4) | 11.1965(4) |
| *b* / Å | 4.4214(2) | 8.3066(3) |
| *c* / Å | 40.079(2) | 24.7197(7) |
| *α* / ° | 90 | 90 |
| *β* / ° | 96.017(5) | 90 |
| *γ* / ° | 90 | 90 |
| *V* / Å^3^ | 1515.95(13) | 2299.05(13) |
| *Z* | 2 | 4 |
| *D*_calc_ / g cm^−3^ | 2.637 | 1.832 |
| *λ*(Mo*K*_α_) / Å | 0.71073 | 0.71073 |
| *T* / K | 295 | 295 |
| Crystal size / mm^3^ | 0.51 x 0.16 x 0.08 | 0.40 x 0.34 x 0.26 |
| *μ* / mm^−1^ | 6.202 | 2.768 |
| *F*(000) | 1084 | 1224 |
| Refl. collected/unique | 15373 / 4412 | 18111 / 3851 |
| Parameters/restraints | 173 / 0 | 137 / 0 |
| Δ*ρ*_max_ , Δ*ρ*_min_ / e Å^−3^ | 1.151; –0.641 | 1.179; –0.939 |
| *R*[*F*^2^ > 4*σ*(*F*^2^)] | 0.0454 | 0.0327 |
| w*R*(*F*^2^) | 0.1255 | 0.0861 |
| Goodness-of-fit, *S* | 1.091 | 1.049 |

**Table S3.** continued

|  | (**44diMebpy**)(**NBSac**)_2_ | (**66diMebpy**)(**NIS**)_2_ |
| --- | --- | --- |
| Molecular formula | (C_12_H_12_N_2_)(C_7_H_4_BrNO_3_S)_2_ | (C_12_H_12_N_2_)(C_4_H_4_INO_2_)_2_ |
| *M*_r_ | 708.40 | 634.20 |
| Crystal system | triclinic | orthorombic |
| Space group | *P* $\overline{1}$ | *P* *bcn* |
| Crystal data: |  |  |
| *a* / Å | 8.0118(4) | 12.1898(7) |
| *b* / Å | 11.6140(5) | 9.5200(5) |
| *c* / Å | 16.4348(7) | 19.9607(10) |
| *α* / ° | 71.632(4) | 90 |
| *β* / ° | 76.515(4) | 90 |
| *γ* / ° | 78.573(4) | 90 |
| *V* / Å^3^ | 1398.39(12) | 2316.4(2) |
| *Z* | 2 | 4 |
| *D*_calc_ / g cm^−3^ | 1.682 | 1.819 |
| *λ*(Mo*K*_α_) / Å | 0.71073 | 0.71073 |
| *T* / K | 295 | 295 |
| Crystal size / mm^3^ | 0.59 x 0.51 x 0.11 | 0.57 x 0.56 x 0.53 |
| *μ* / mm^−1^ | 3.096 | 2.748 |
| *F*(000) | 708 | 1224 |
| Refl. collected/unique | 25257 / 9358 | 25366 / 3983 |
| Parameters/restraints | 363 / 0 | 137 / 0 |
| Δ*ρ*_max_ , Δ*ρ*_min_ / e Å^−3^ | 0.995; –0.536 | 1.004; –0.558 |
| *R*[*F*^2^ > 4*σ*(*F*^2^)] | 0.0580 | 0.0454 |
| w*R*(*F*^2^) | 0.1395 | 0.1253 |
| Goodness-of-fit, *S* | 1.019 | 0.978 |

**Table S3.** continued

|  | (**66diMebpy**)_2_(**14tfib**) | (**44tBubpy**)(**14tfib**) |
| --- | --- | --- |
| Molecular formula | (C_12_H_12_N_2_)_2_(C_6_F_4_I_2_) | (C_18_H_24_N_2_)(C_6_F_4_I_2_) |
| *M*_r_ | 770.33 | 670.25 |
| Crystal system | triclinic | triclinic |
| Space group | *P* $\overline{1}$ | *P* $\overline{1}$ |
| Crystal data: |  |  |
| *a* / Å | 6.9031(2) | 5.9330(2) |
| *b* / Å | 9.2688(3) | 10.5414(4) |
| *c* / Å | 12.4053(4) | 10.8185(4) |
| *α* / ° | 99.511(2) | 110.547(3) |
| *β* / ° | 99.430(2) | 91.481(3) |
| *γ* / ° | 101.735(2) | 92.245(3) |
| *V* / Å^3^ | 750.27(4) | 632.52(4) |
| *Z* | 1 | 1 |
| *D*_calc_ / g cm^−3^ | 1.705 | 1.760 |
| *λ*(Mo*K*_α_) / Å | 0.71073 | 0.71073 |
| *T* / K | 295 | 295 |
| Crystal size / mm^3^ | 0.51 x 0.48 x 0.24 | 0.60 x 0.48 x 0.19 |
| *μ* / mm^−1^ | 2.146 | 2.529 |
| *F*(000) | 374 | 324 |
| Refl. collected/unique | 14597 / 5029 | 12379 / 4267 |
| Parameters/restraints | 184 / 0 | 179 / 0 |
| Δ*ρ*_max_ , Δ*ρ*_min_ / e Å^−3^ | 1.163; –0.752 | 0.471; –0.839 |
| *R*[*F*^2^ > 4*σ*(*F*^2^)] | 0.0444 | 0.0398 |
| w*R*(*F*^2^) | 0.1291 | 0.1168 |
| Goodness-of-fit, *S* | 1.038 | 1.028 |

**Table S3.** continued

|  | (**22biq**)(**14tfib**) |
| --- | --- |
| Molecular formula | (C_18_H_12_N_2_)(C_6_F_4_I_2_) |
| *M*_r_ | 658.16 |
| Crystal system | triclinic |
| Space group | *P* $\overline{1}$ |
| Crystal data: |  |
| *a* / Å | 5.9747(2) |
| *b* / Å | 6.06750(10) |
| *c* / Å | 14.8555(4) |
| *α* / ° | 92.708(2) |
| *β* / ° | 91.917(2) |
| *γ* / ° | 96.526(2) |
| *V* / Å^3^ | 534.04(2) |
| *Z* | 1 |
| *D*_calc_ / g cm^−3^ | 2.046 |
| *λ*(Mo*K*_α_) / Å | 0.71073 |
| *T* / K | 295 |
| Crystal size / mm^3^ | 0.56 x 0.44 x 0.26 |
| *μ* / mm^−1^ | 2.994 |
| *F*(000) | 312 |
| Refl. collected/unique | 13827 / 3597 |
| Parameters/restraints | 145 / 0 |
| Δ*ρ*_max_ , Δ*ρ*_min_ / e Å^−3^ | 0.746; –0.905 |
| *R*[*F*^2^ > 4*σ*(*F*^2^)] | 0.0371 |
| w*R*(*F*^2^) | 0.1089 |
| Goodness-of-fit, *S* | 1.084 |

**
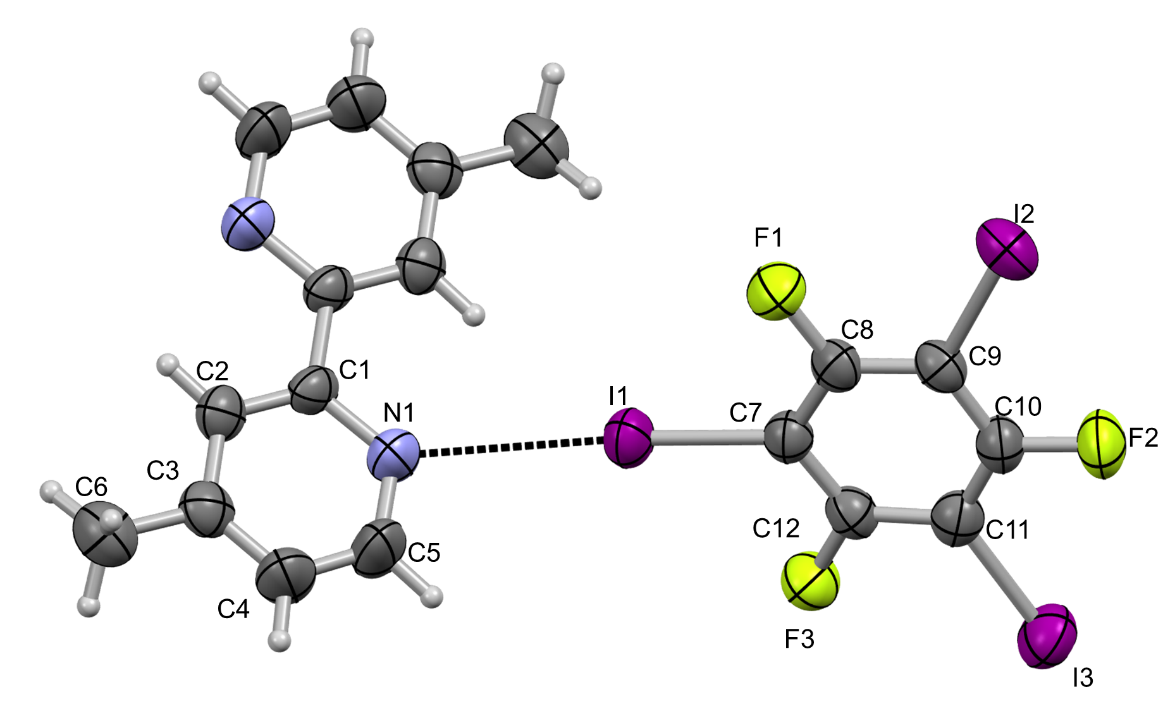
**

**Figure S1.** Molecular structure of (**44diMebpy**)(**135tfib**)_2_ showing the atom-labeling scheme. Displacement ellipsoids are drawn at the 50 % probability level, and H atoms are shown as small spheres of arbitrary radius.


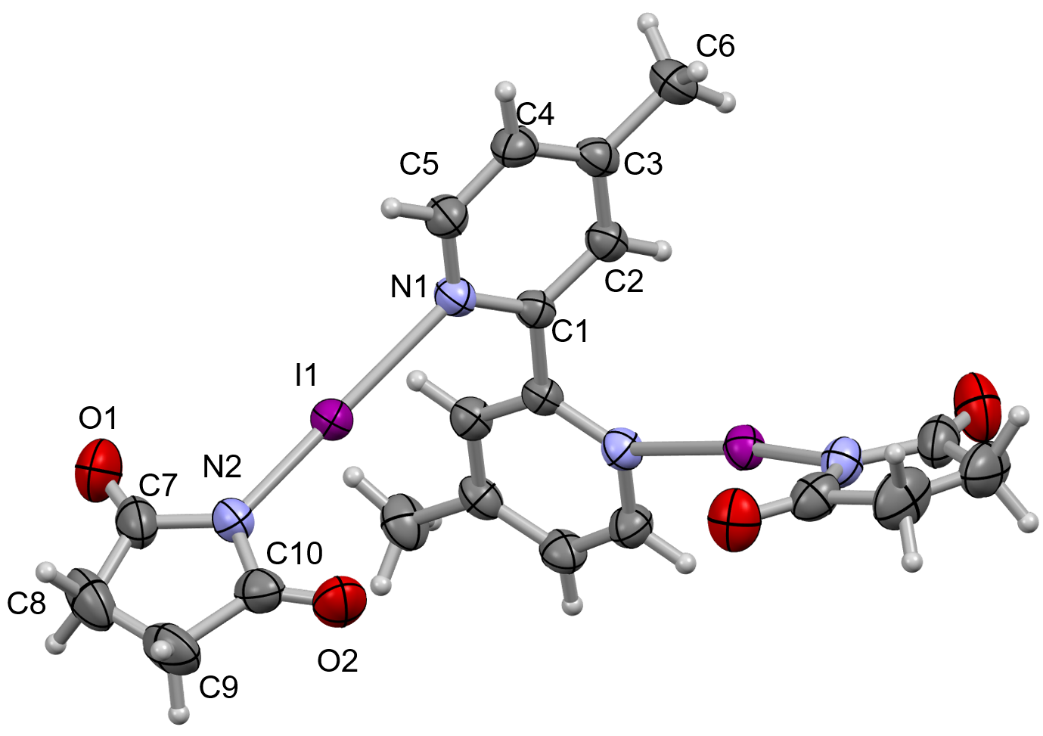


**Figure S2.** Molecular structure of (**44diMebpy**)(**NIS**)_2_ showing the atom-labeling scheme. Displacement ellipsoids are drawn at the 50 % probability level, and H atoms are shown as small spheres of arbitrary radius.


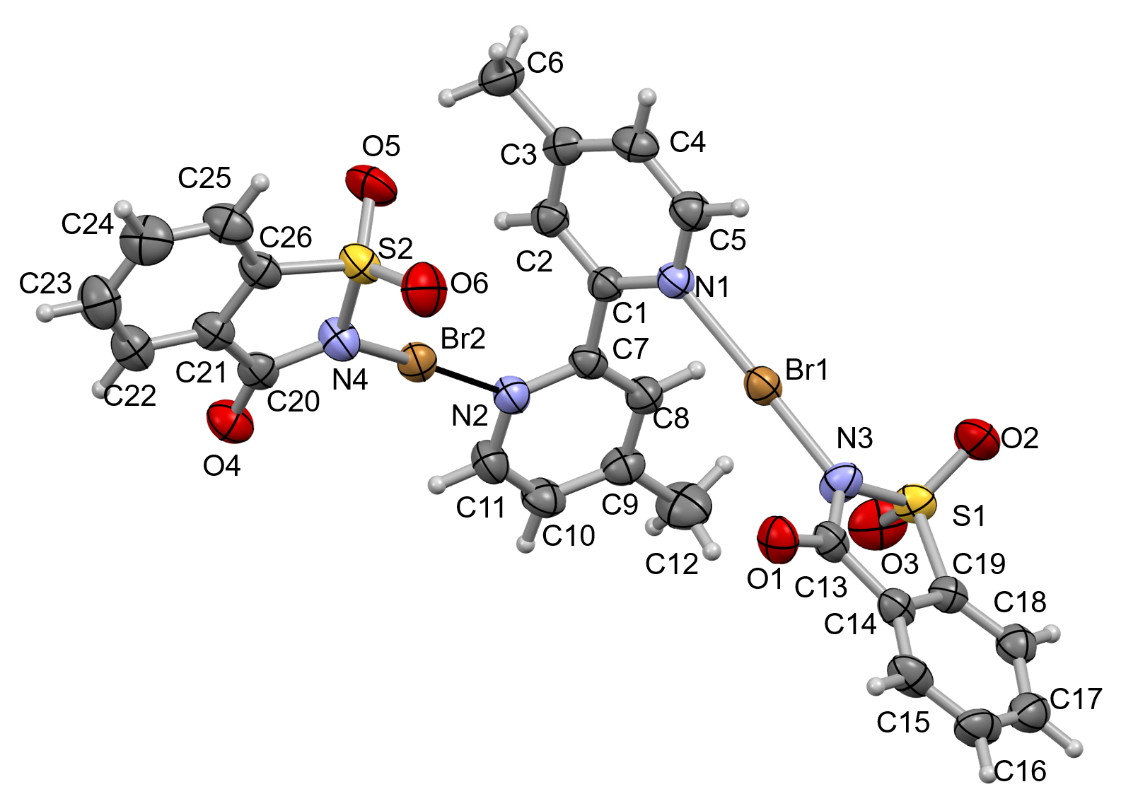


**Figure S3.** Molecular structure of (**44diMebpy**)(**NBSac**)_2_ showing the atom-labeling scheme. Displacement ellipsoids are drawn at the 50 % probability level, and H atoms are shown as small spheres of arbitrary radius.


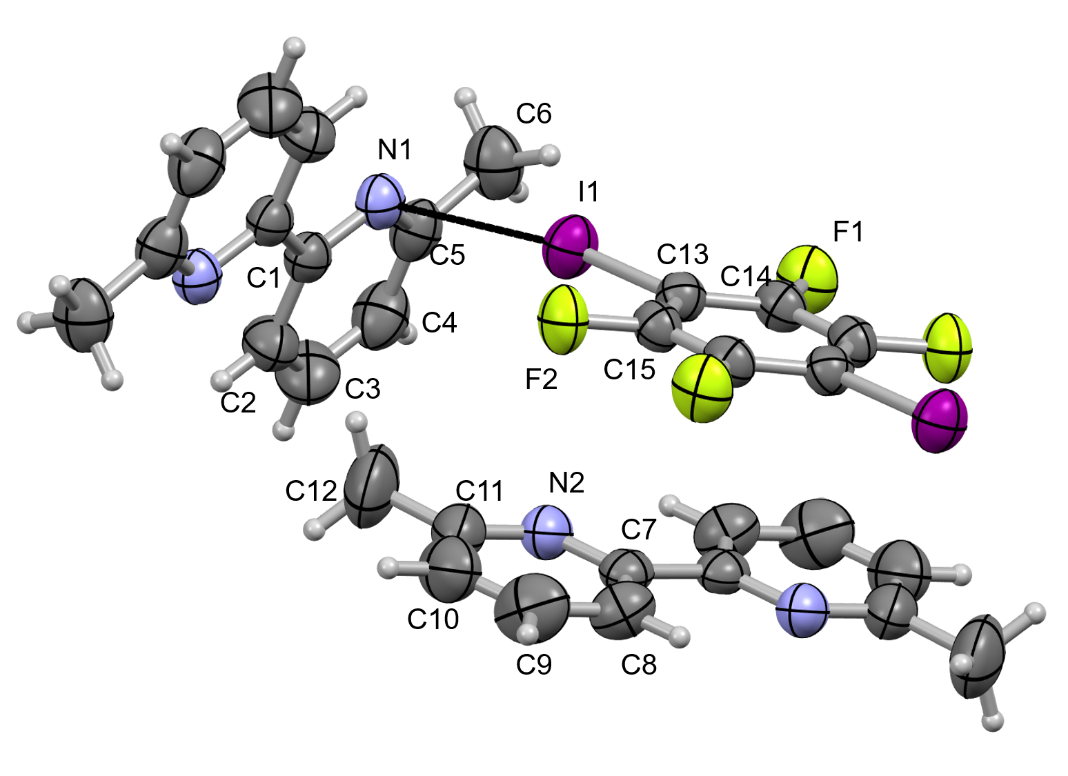


**Figure S4.** Molecular structure of (**66diMebpy**)_2_(**14tfib**) showing the atom-labeling scheme. Displacement ellipsoids are drawn at the 50 % probability level, and H atoms are shown as small spheres of arbitrary radius.


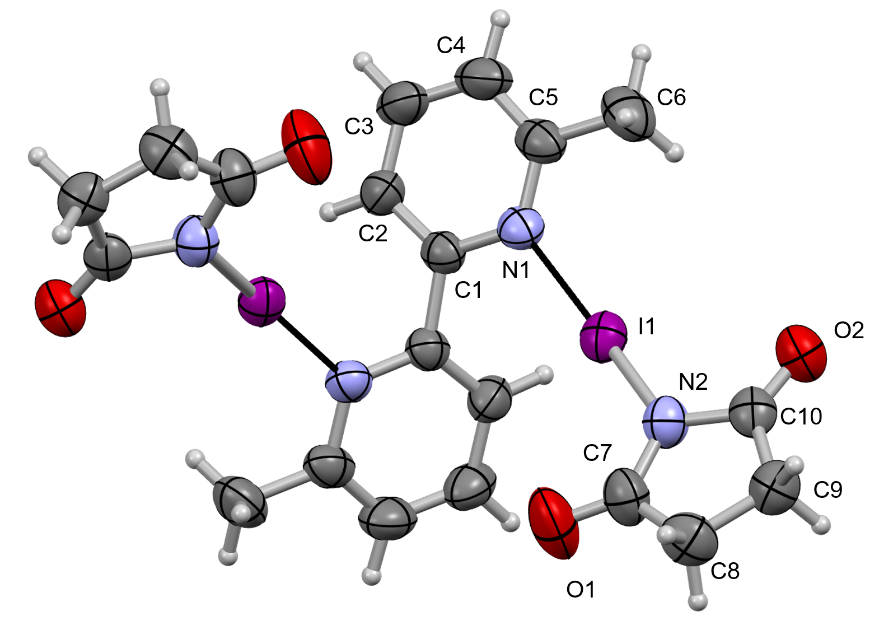


**Figure S5.** Molecular structure of (**66diMebpy**)(**NIS**)_2_ showing the atom-labeling scheme. Displacement ellipsoids are drawn at the 50 % probability level, and H atoms are shown as small spheres of arbitrary radius.


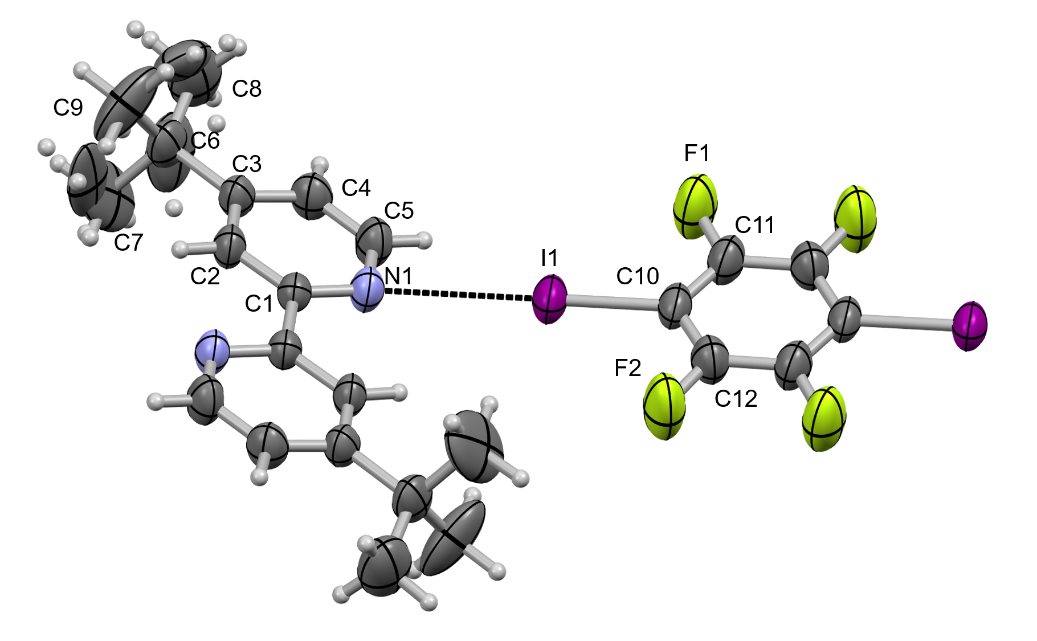


**Figure S6.** Molecular structure of (**44tBuMebpy**)(**14tfib**) showing the atom-labeling scheme. Displacement ellipsoids are drawn at the 50 % probability level, and H atoms are shown as small spheres of arbitrary radius.


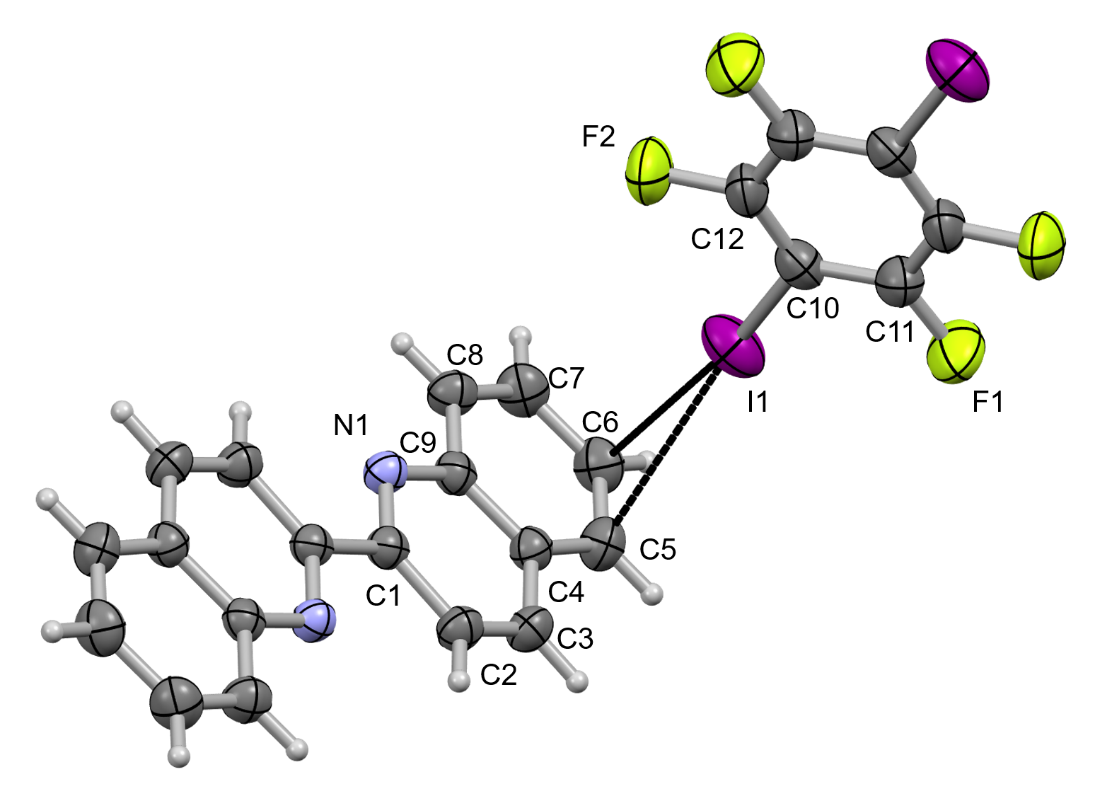


**Figure S7.** Molecular structure of (**22biq**)(**14tfib**) showing the atom-labeling scheme. Displacement ellipsoids are drawn at the 50 % probability level, and H atoms are shown as small spheres of arbitrary radius.


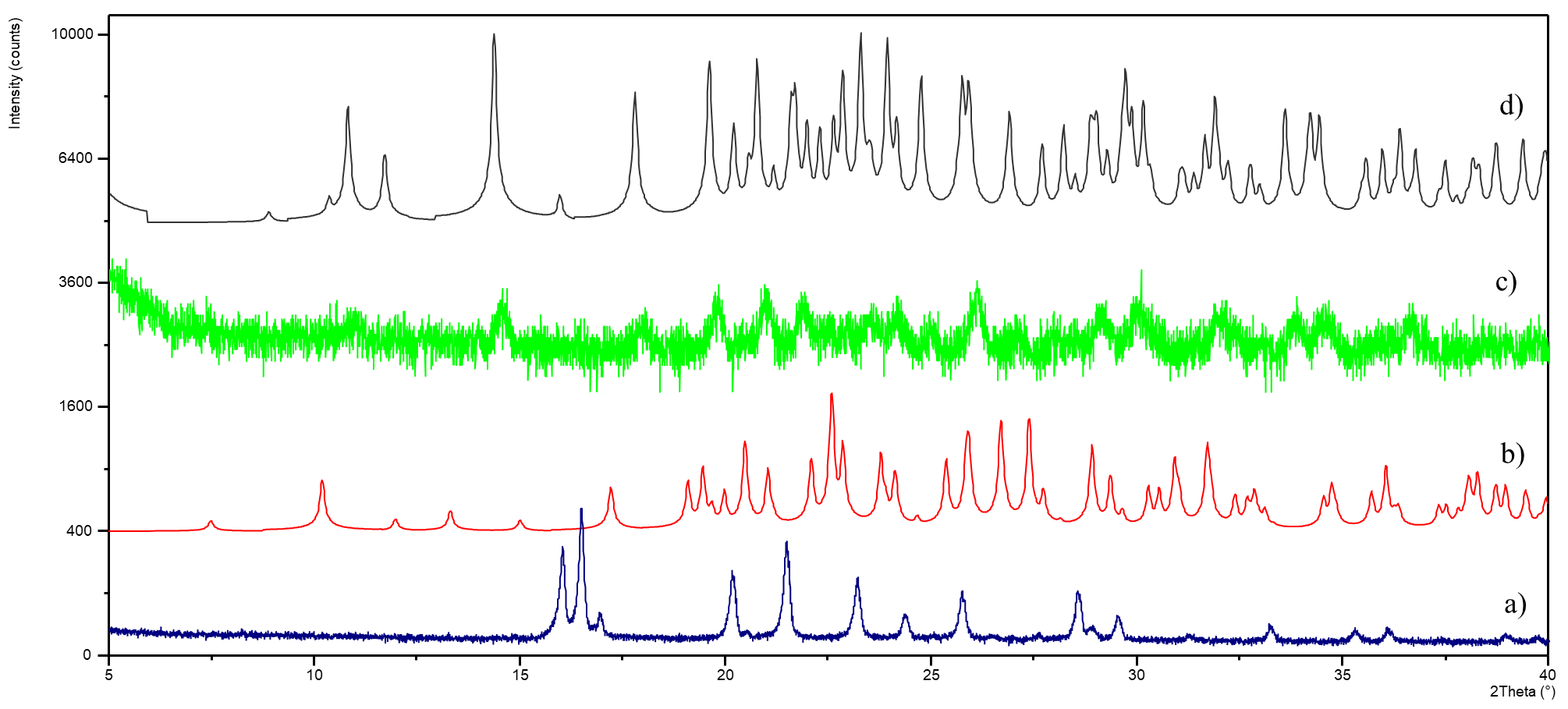


**Figure S8.** PXRD patterns of: a) **44diMebpy**, b) **135tfib**, c) product obtained by grinding **44diMebpy** and **135tfib** in a 1:1 stoichiometric ratio d) calculated pattern from single crystal data.


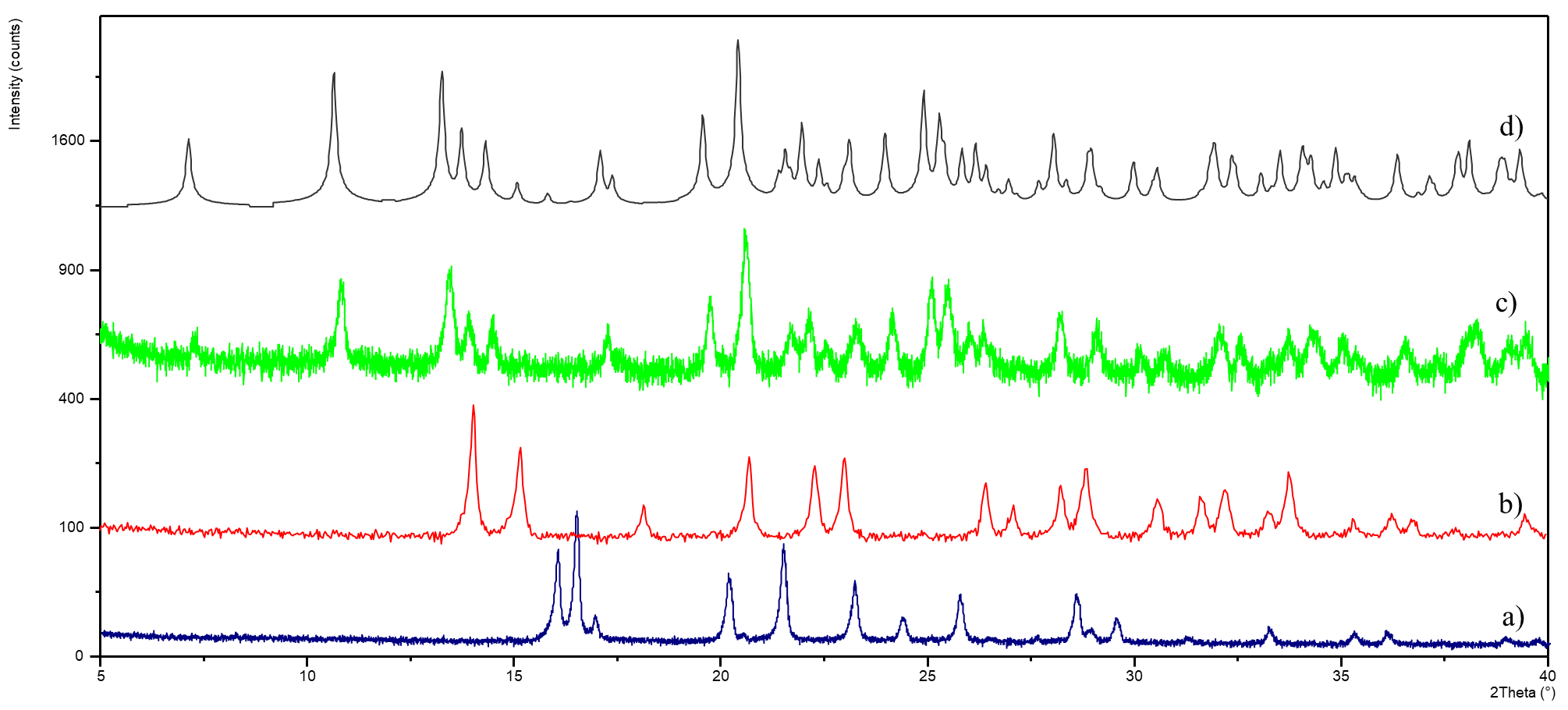


**Figure S9.** PXRD patterns of: a) **44diMebpy**, b) **NIS**, c) product obtained by grinding **44diMebpy** and **NIS** in a 1:2 stoichiometric ratio d) calculated pattern from single crystal data.


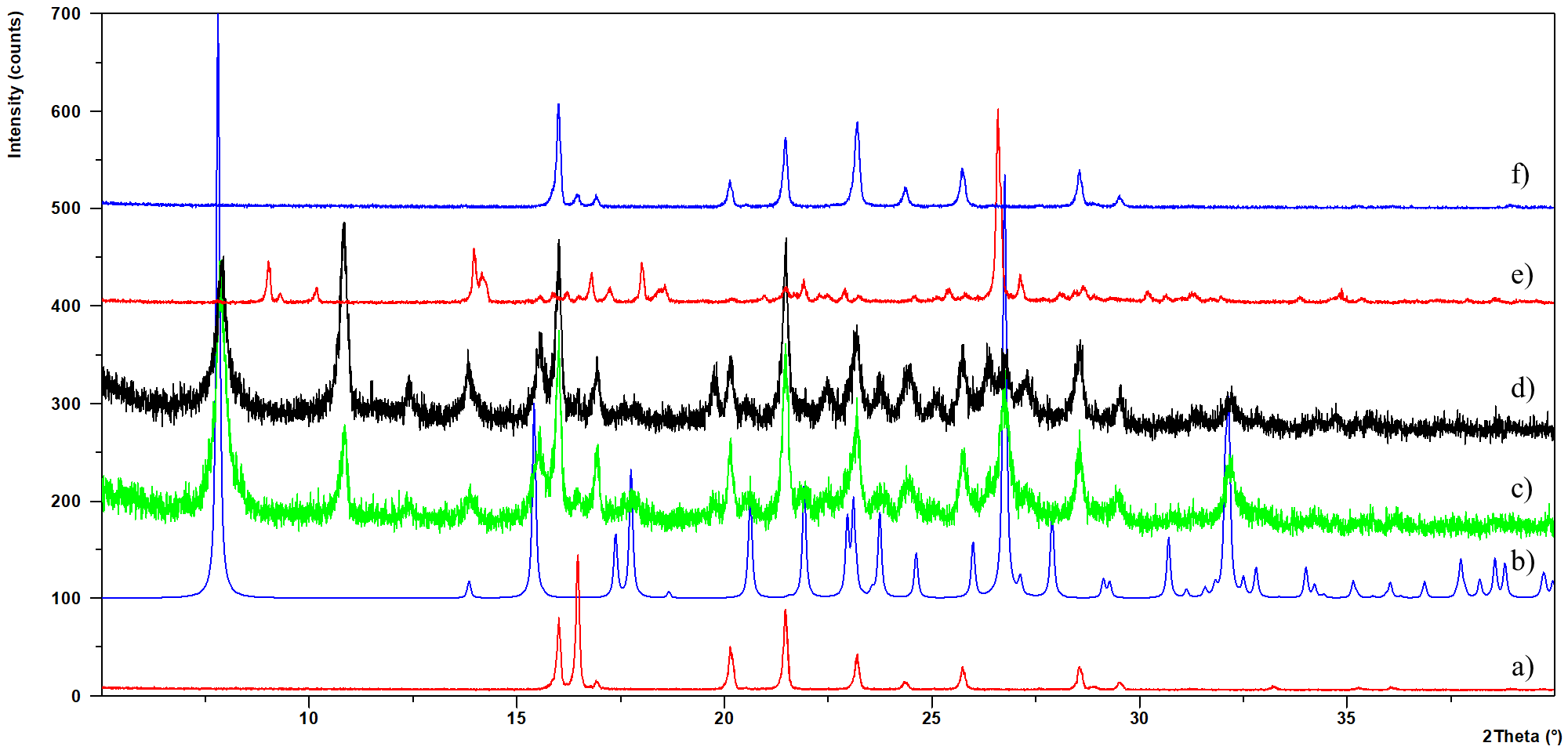


**Figure S10.** PXRD patterns of: a) **44diMebpy**, b) **NBF**, c) product obtained by grinding **44diMebpy** and **NBF** in a 1:1 stoichiometric ratio for 10 minutes, d) product obtained by grinding **44diMebpy** and **NBF** in a 1:1 stoichiometric ratio for 30 minutes, e) bulk product obtained by dissolving **44diMebpy** and **NBF** in a 1:1 stoichiometric ratio in tetrahydrofuran, f) bulk product obtained by dissolving **44diMebpy** and **NBF** in a 1:1 stoichiometric ratio in acetonitrile.


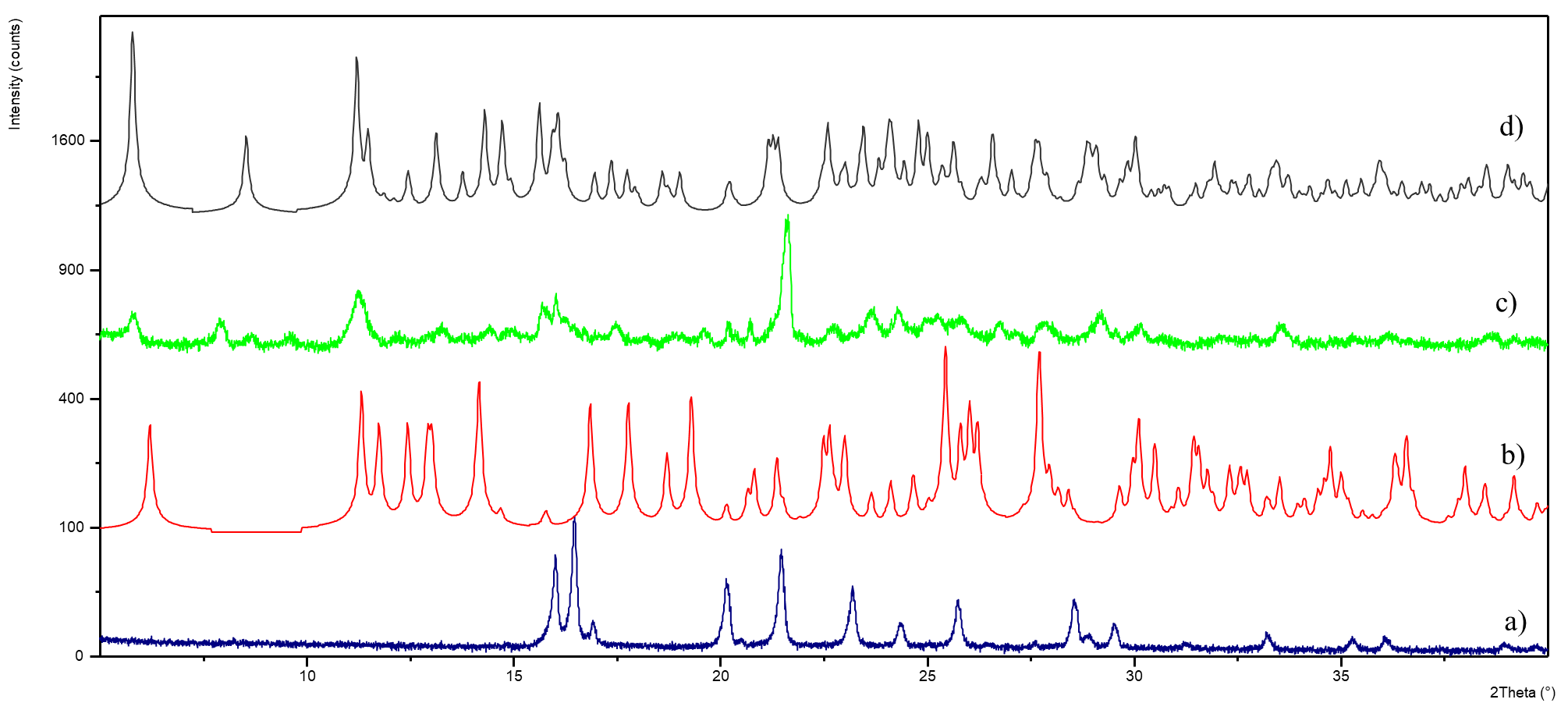


**Figure S11.** PXRD patterns of: a) **44diMebpy**, b) **NBSac**, c) product obtained by grinding **44diMebpy** and **NBSac** in a 1:2 stoichiometric ratio d) calculated pattern from single crystal data.


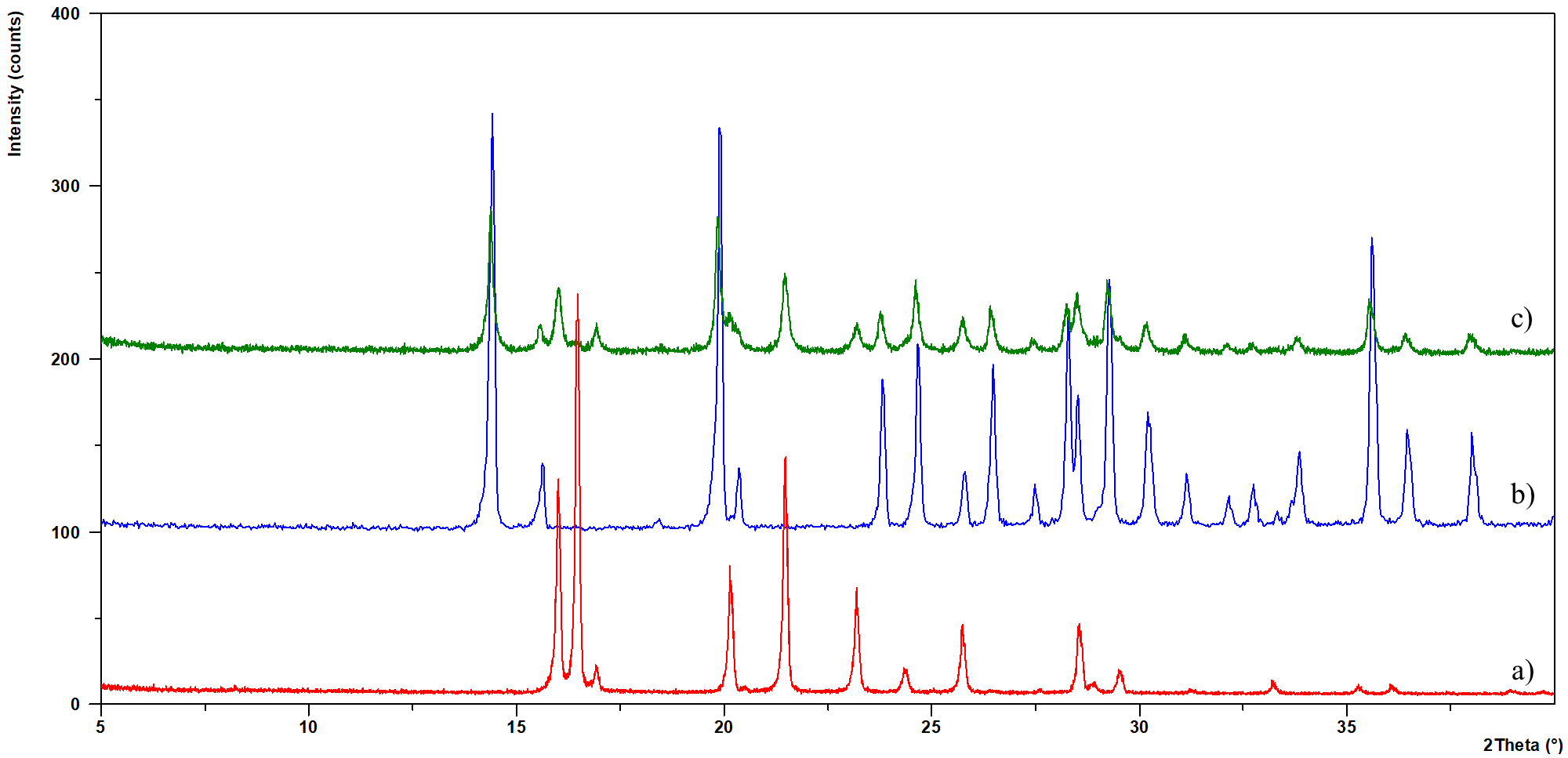


**Figure S12.** PXRD patterns of: a) **44diMebpy**, b) **NBS**, c) product obtained by grinding **44diMebpy** and **NBS** in a 1:1 stoichiometric ratio.


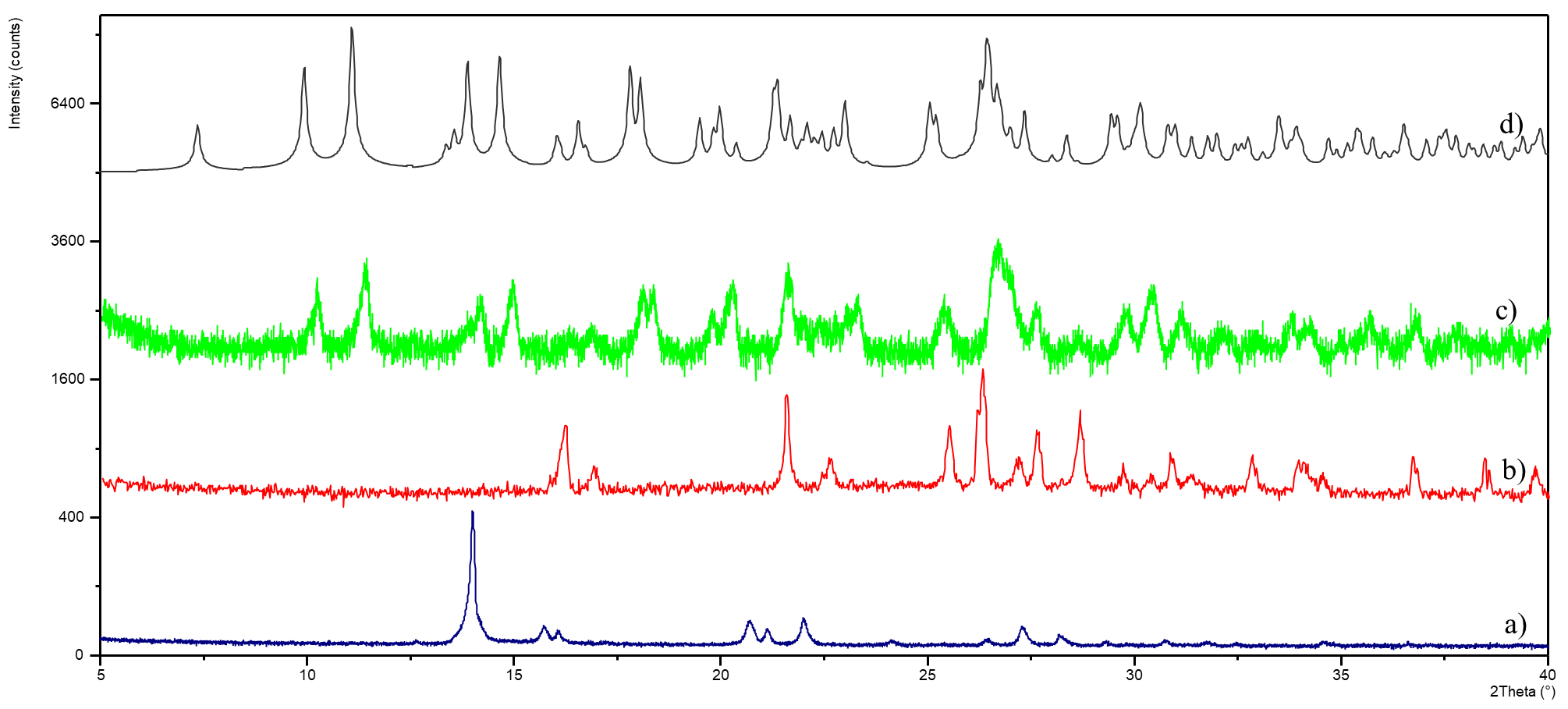


**Figure S13.** PXRD patterns of: a) **66diMebpy**, b) **14tfib**, c) product obtained by grinding **66diMebpy** and **14tfib** in a 2:1 stoichiometric ratio d) calculated pattern from single crystal data.


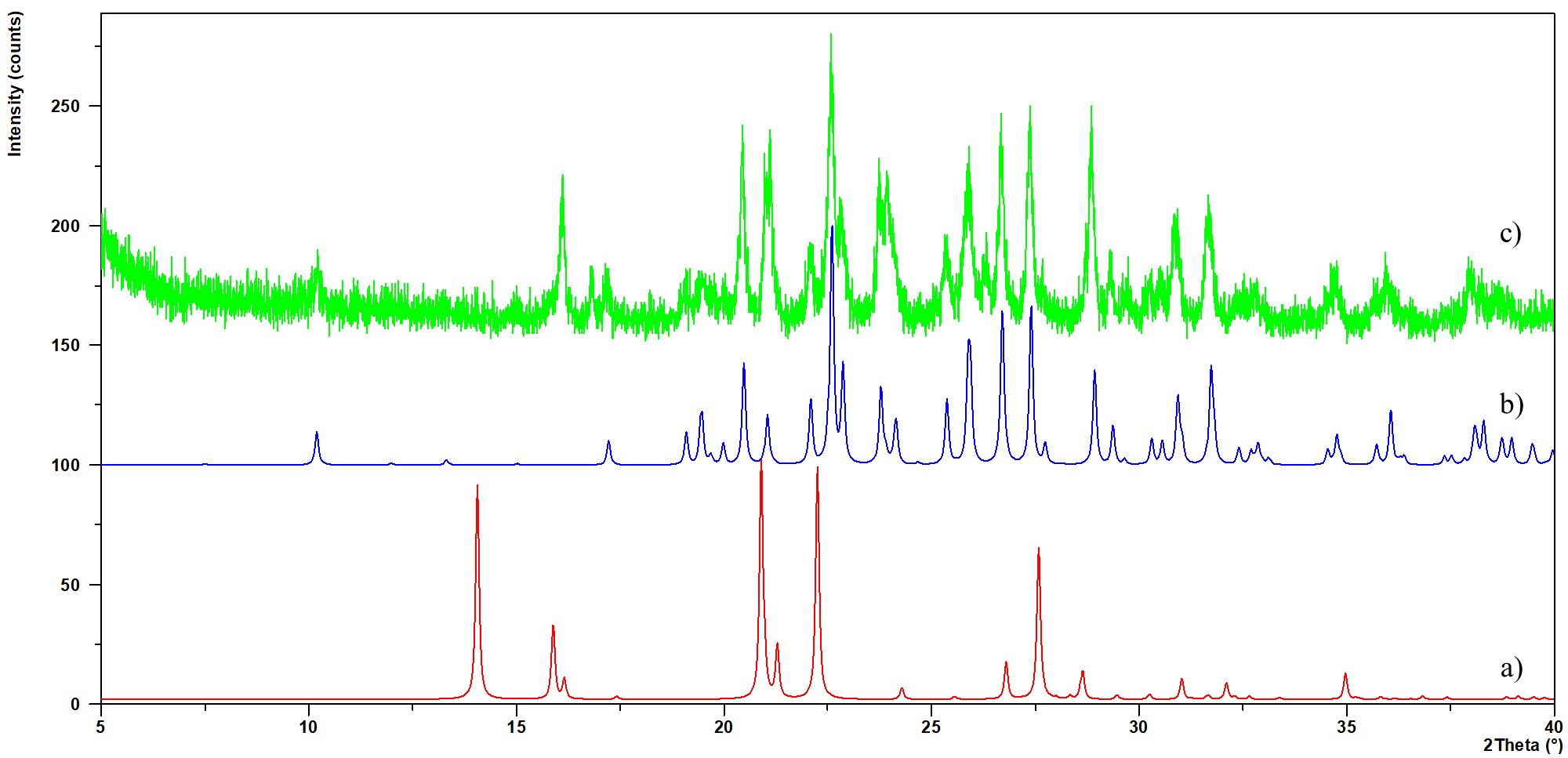


**Figure S14.** PXRD patterns of: a) **66diMebpy** calculated from single crystal data, b) **135tfib** calculated from single crystal data, c) product obtained by grinding **66diMebpy** and **135tfib** in a 2:1 stoichiometric ratio.


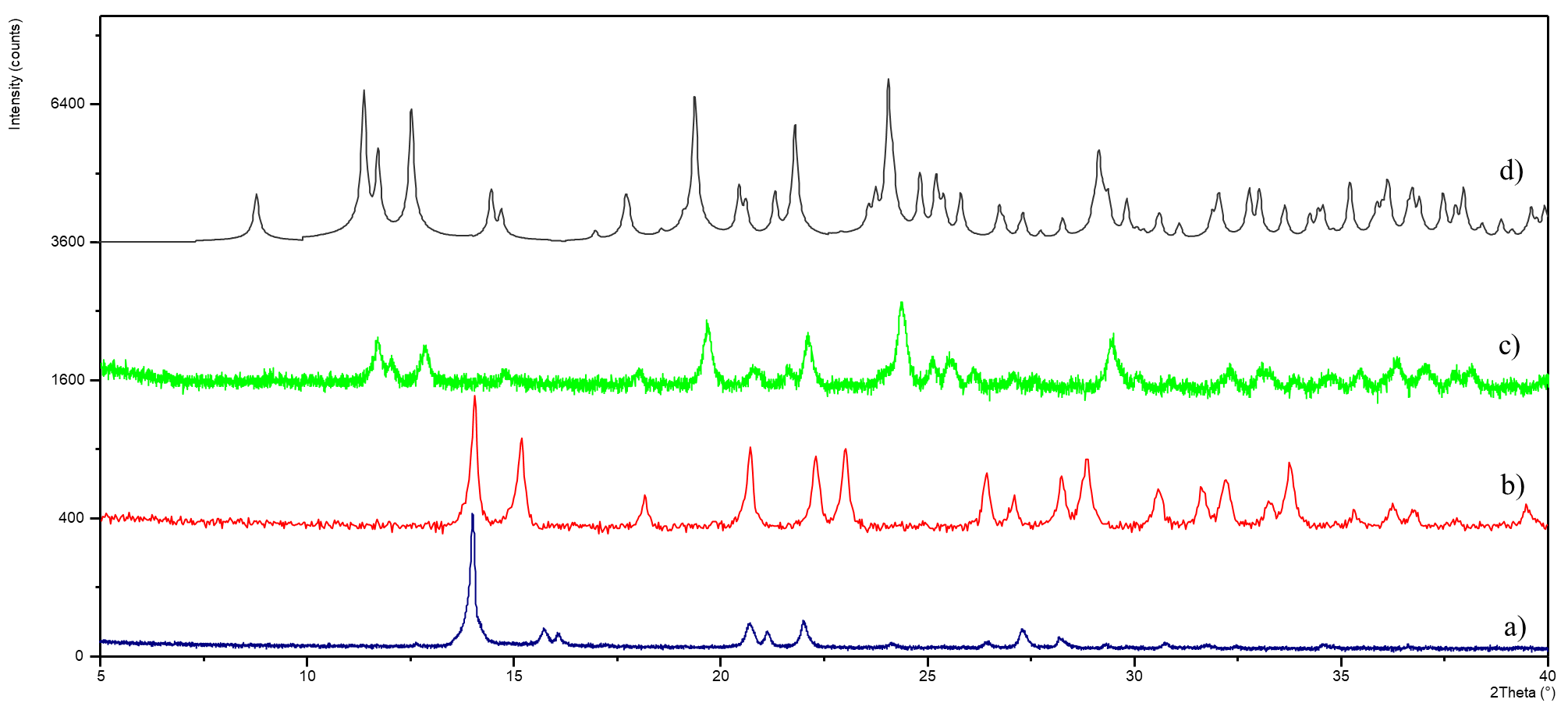


**Figure S15.** PXRD patterns of: a) **66diMebpy**, b) **NIS**, c) product obtained by grinding **66diMebpy** and **NIS** in a 1:2 stoichiometric ratio d) calculated pattern from single crystal data.


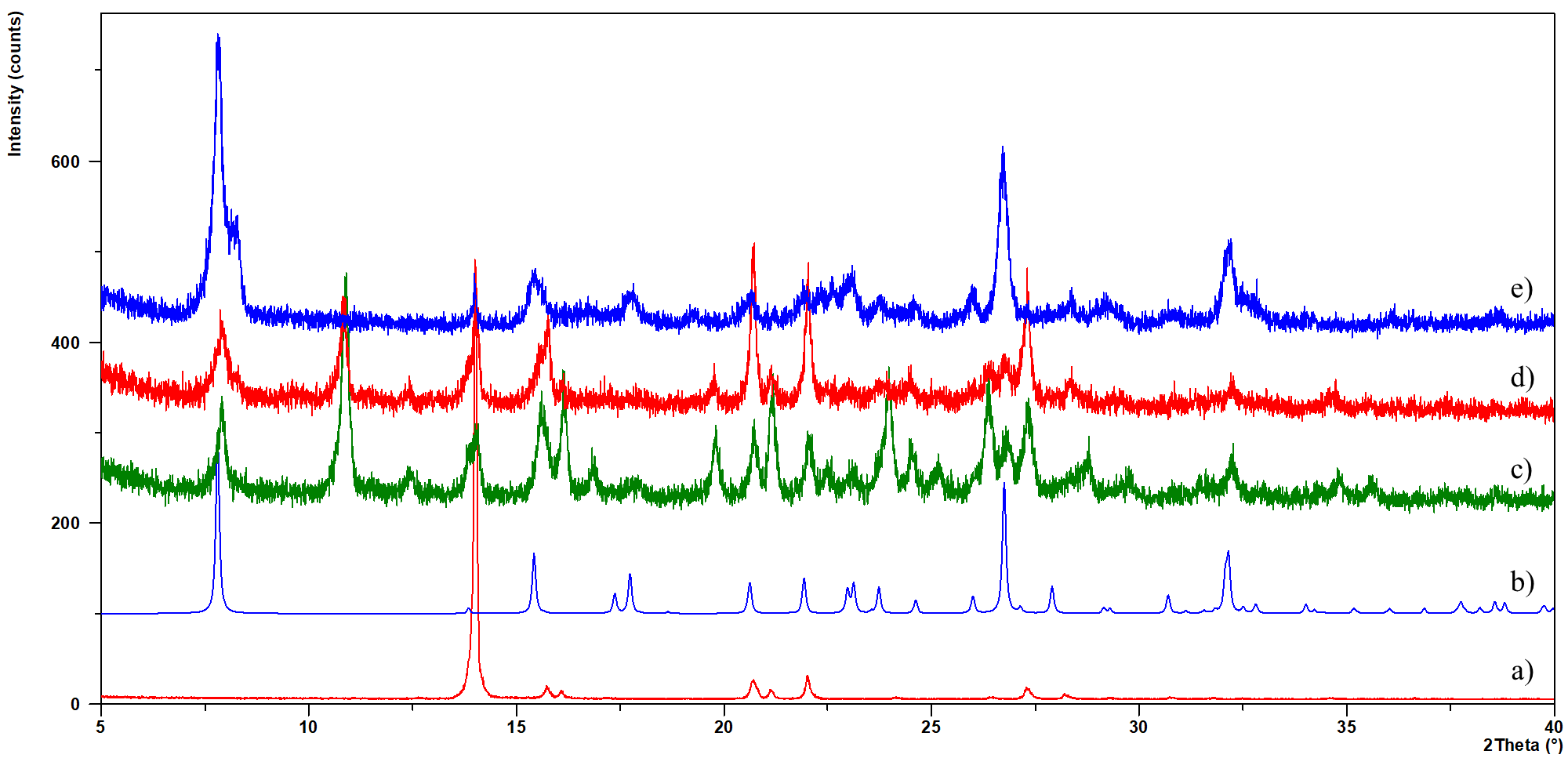


**Figure S16.** PXRD patterns of: a) **66diMebpy**, b) **NBF**, c) product obtained by grinding **66diMebpy** and **NBF** in a 1:1 stoichiometric ratio for 10 minutes, d) product obtained by grinding **66diMebpy** and **NBF** in a 1:1 stoichiometric ratio for 30 minutes, e) bulk product obtained by dissolving **66diMebpy** and **NBF** in a 1:1 stoichiometric ratio in acetonitrile.


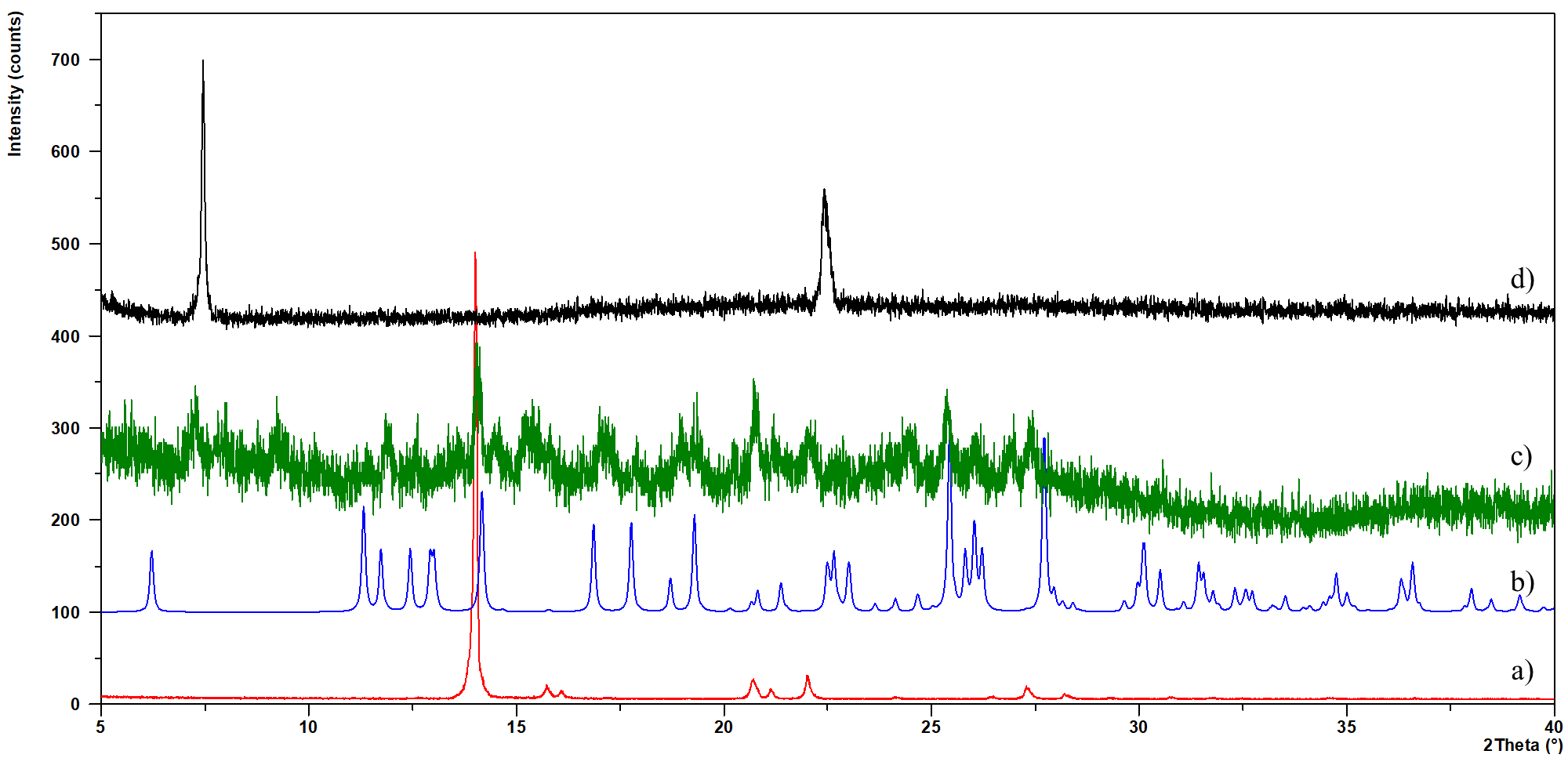


**Figure S17.** PXRD patterns of: a) **66diMebpy**, b) **NBSac**, c) product obtained by grinding **66diMebpy** and **NBSac** in a 1:1 stoichiometric ratio, d) bulk product obtained by dissolving **66diMebpy** and **NBSac** in a 1:1 stoichiometric ratio in dichloromethane.


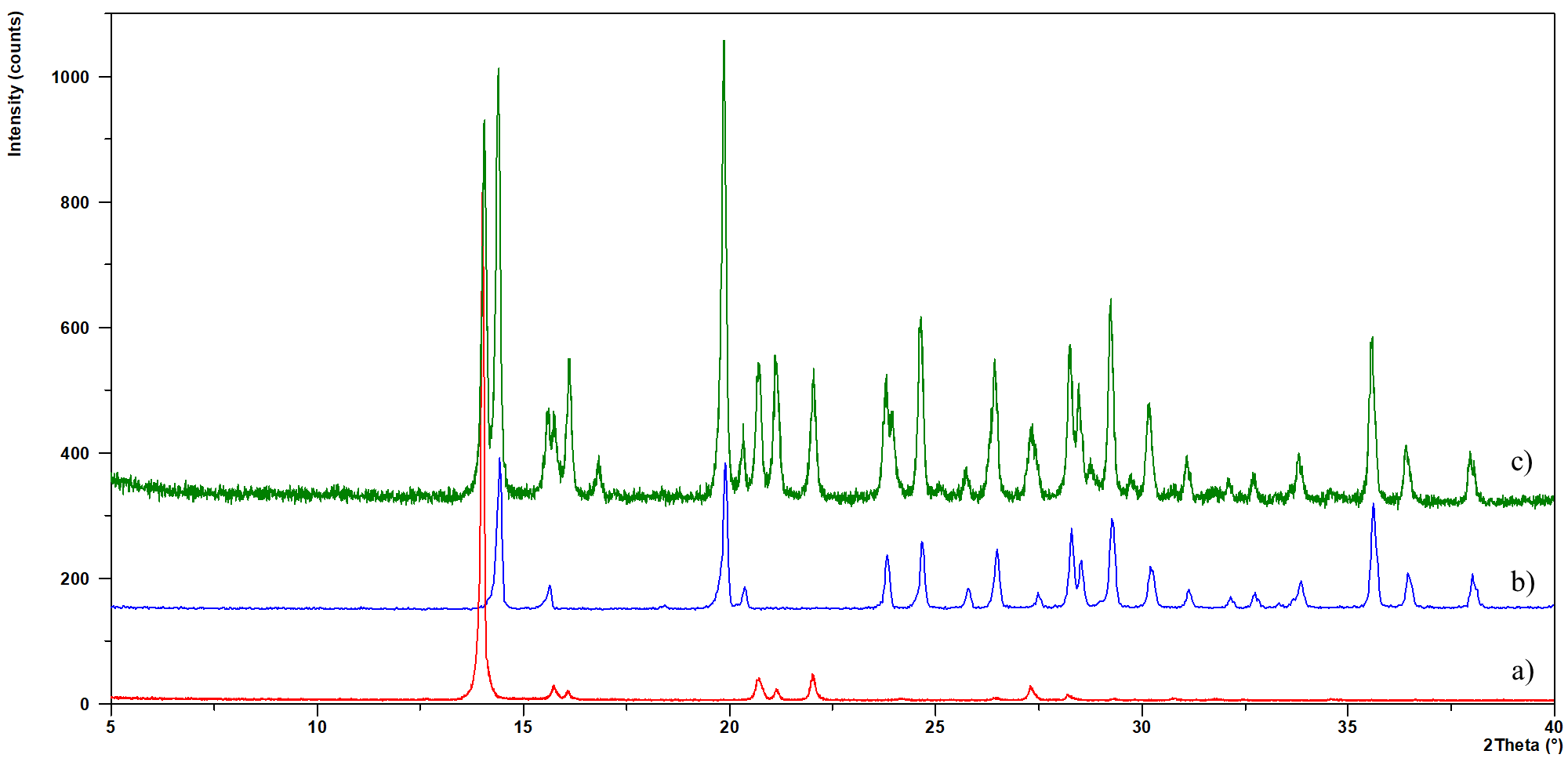


**Figure S18.** PXRD patterns of: a) **66diMebpy**, b) **NBS**, c) product obtained by grinding **66diMebpy** and **NBS** in a 1:1 stoichiometric ratio.


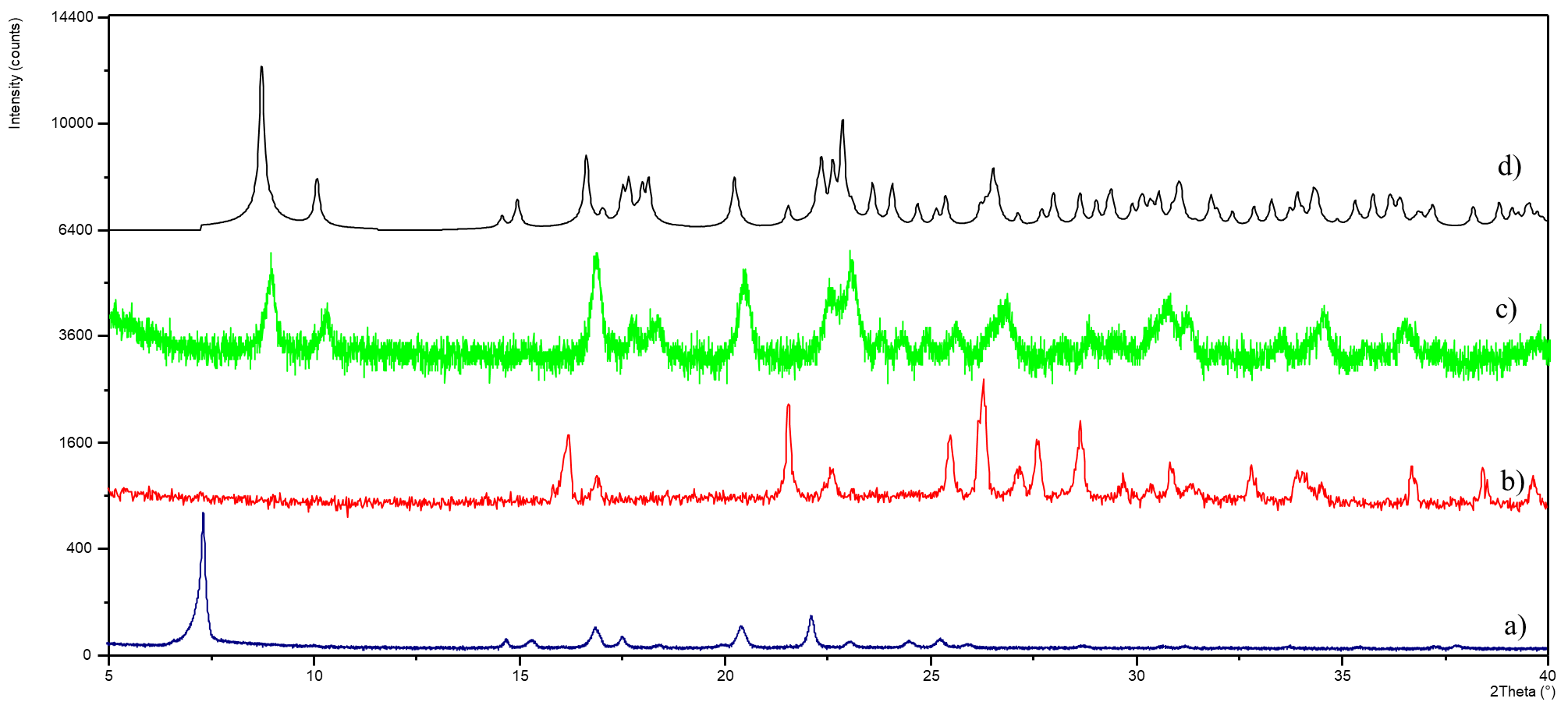


**Figure S19.** PXRD patterns of: a) **44tBubpy**, b) **14tfib**, c) product obtained by grinding **44tBubpy** and **14tfib** in a 1:1 stoichiometric ratio, d) calculated pattern from single crystal data.


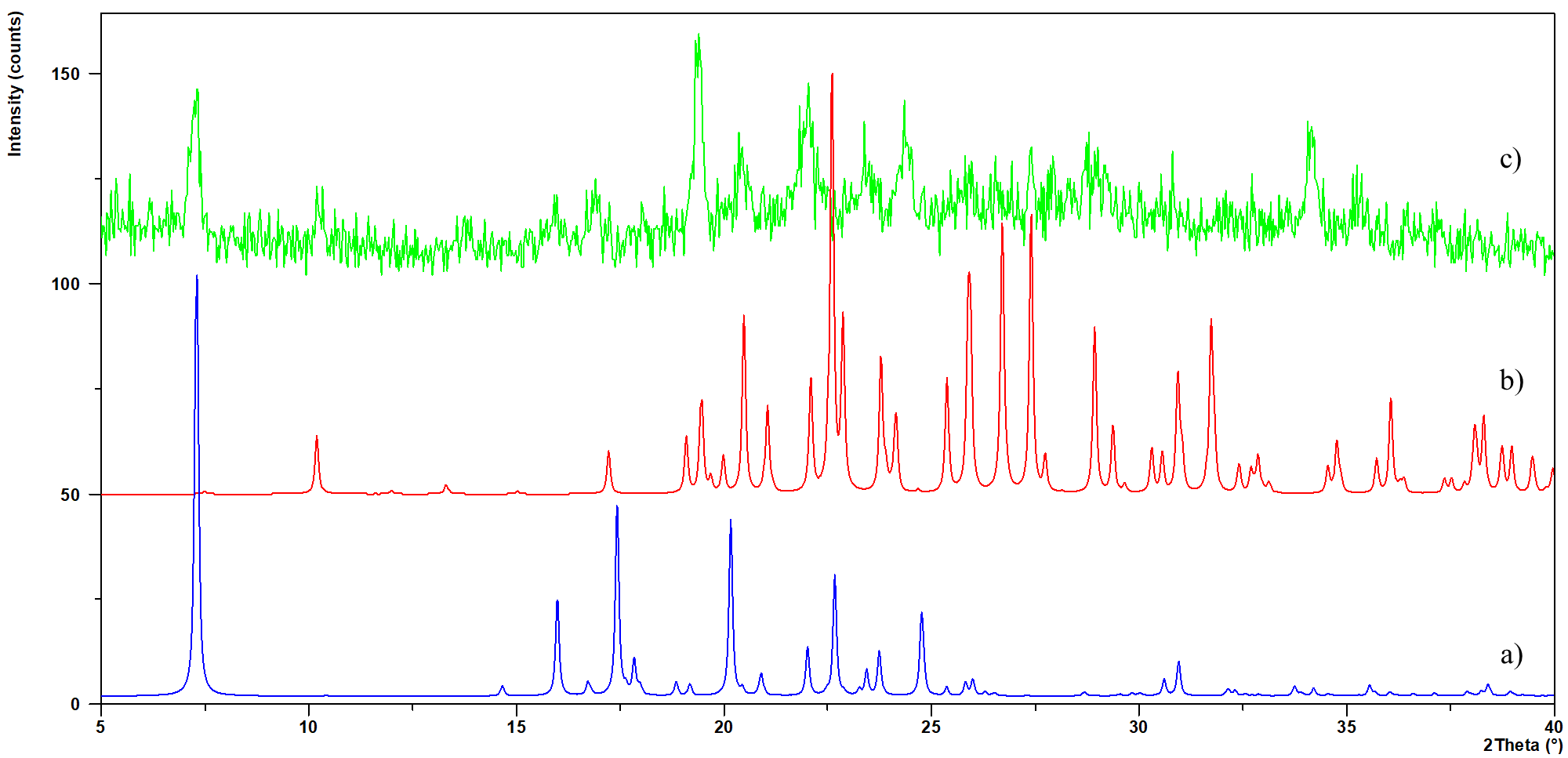


**Figure S20.** PXRD patterns of: a) **44tBubpy** calculated from single crystal data, b) **135tfib** calculated from single crystal data, c) product obtained by grinding **44tBubpy** and **135tfib** in a 1:1 stoichiometric ratio.


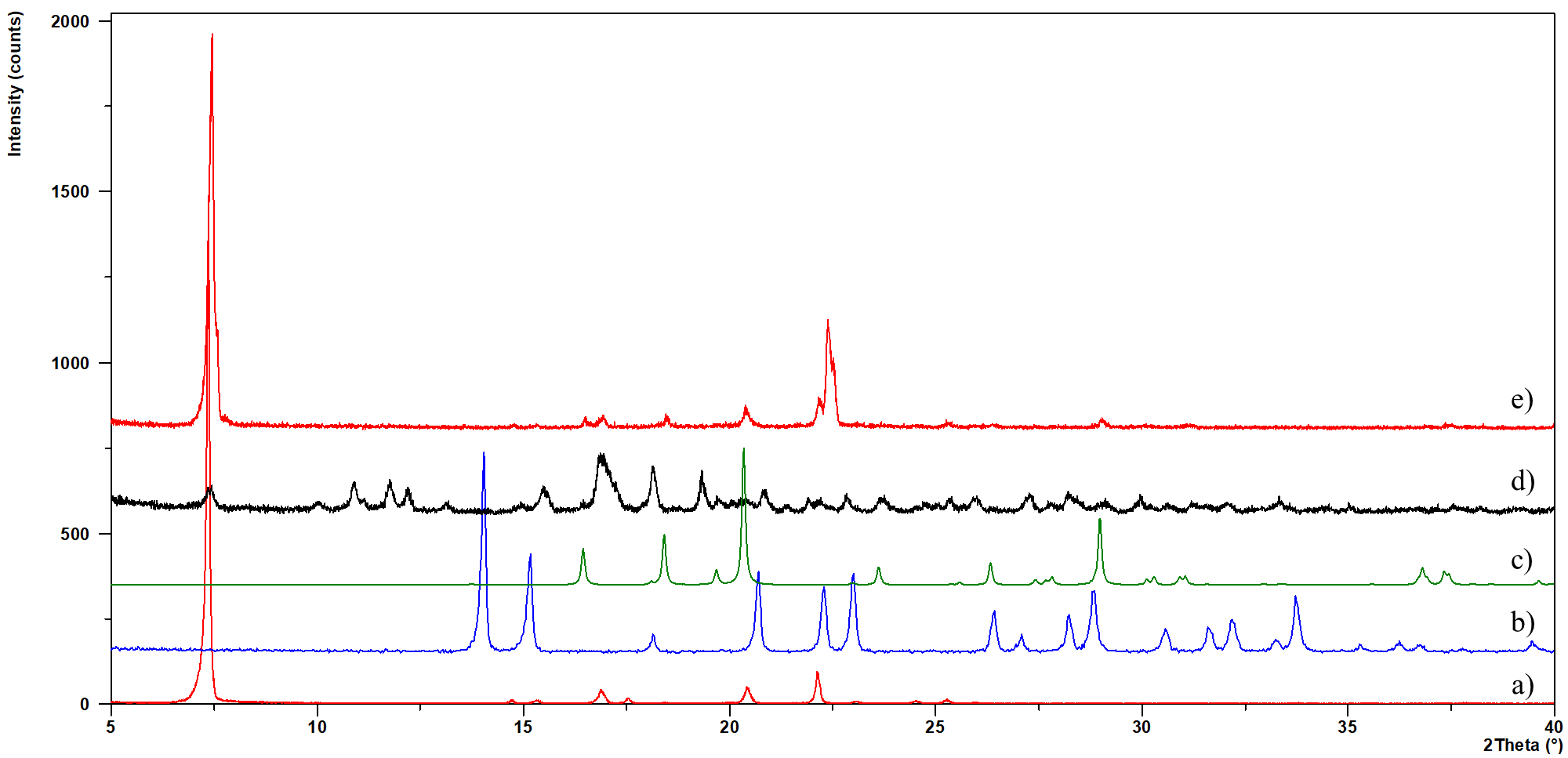


**Figure S21.** PXRD patterns of: a) **44tBubpy** calculated from single crystal data, refcode MUBJOJ, b) **NIS**, c) **NHS** calculated from single crystal data, refcode SUCCIN, d) product obtained by grinding **44tBubpy** and **NIS** in a 1:1 stoichiometric ratio, e) product obtained by dissolving **44tBubpy** and **NIS** in a 1:1 stoichiometric ratio in tetrahydrofuran.


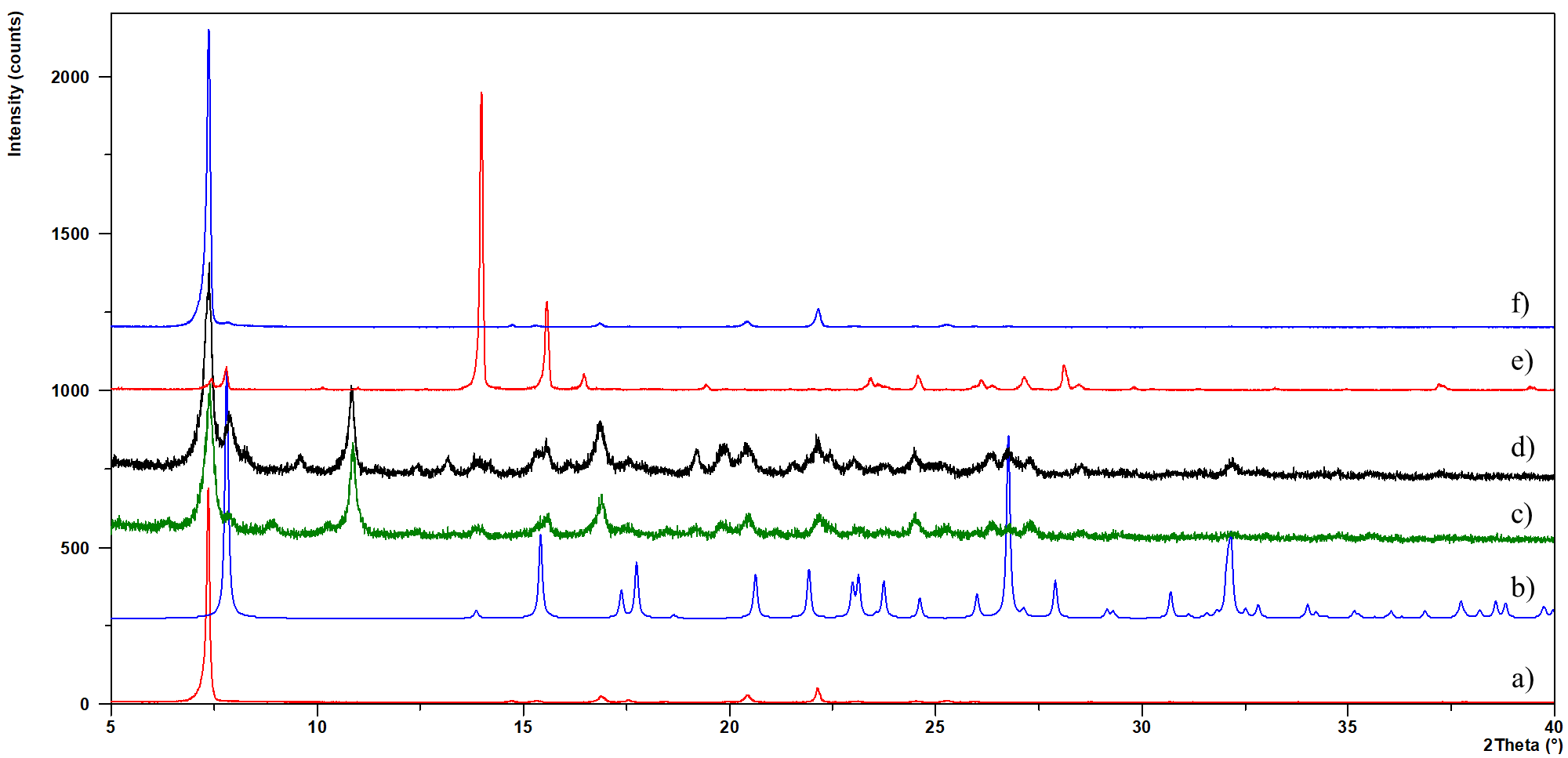


**Figure S22.** PXRD patterns of: a) **44tBubpy** calculated from single crystal data, refcode MUBJOJ, b) **NBF**, c) product obtained by grinding **44tBubpy** and **NBF** in a 1:1 stoichiometric ratio for 10 minutes, d) product obtained by grinding **44tBubpy** and **NBF** in a 1:1 stoichiometric ratio for 30 minutes, e) product obtained by dissolving **44tBubpy** and **NBF** in a 1:1 stoichiometric ratio in tetrahydrofuran, f) product obtained by dissolving **44tBubpy** and **NBF** in a 1:1 stoichiometric ratio in acetonitrile.


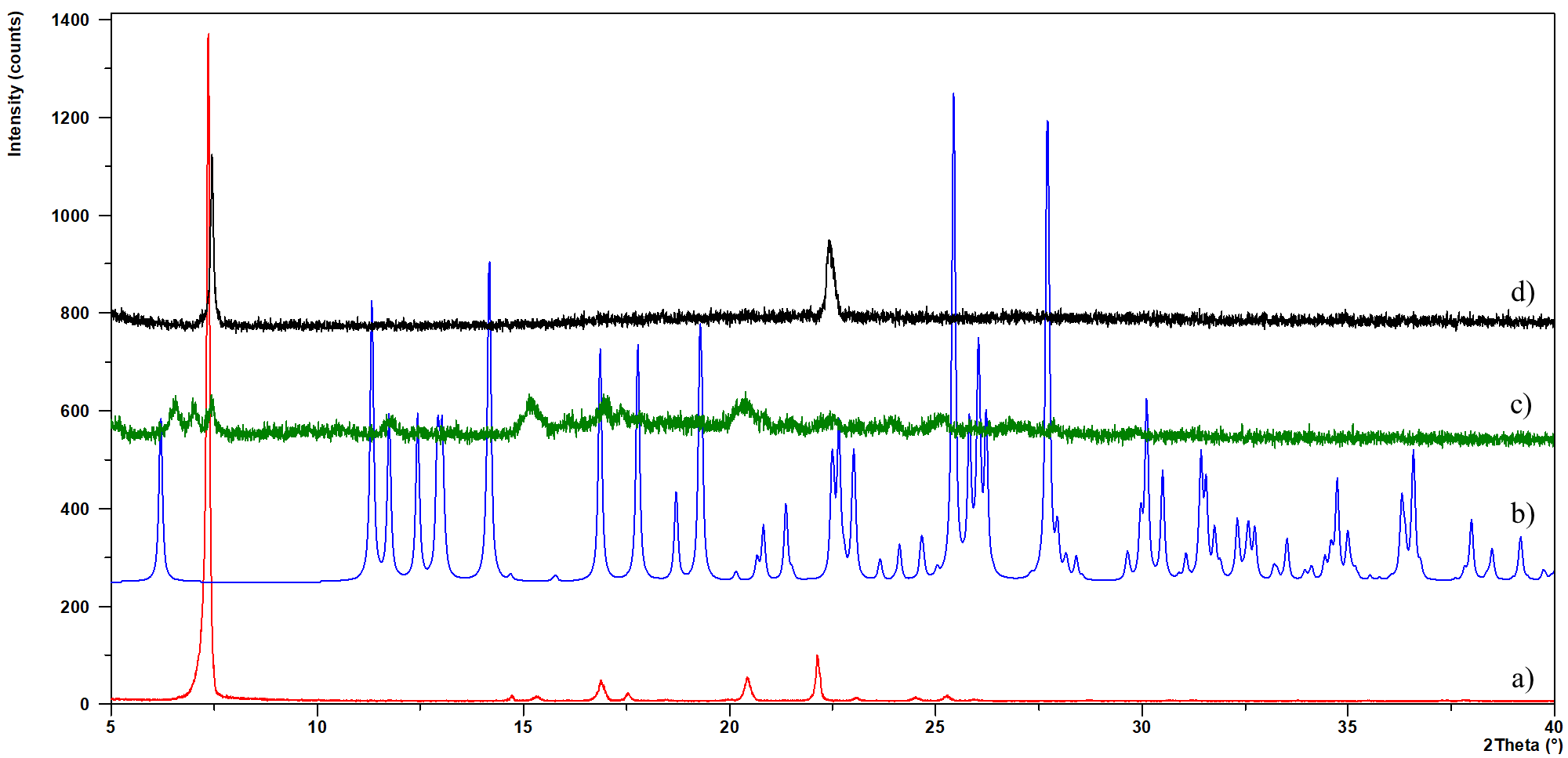


**Figure S23.** PXRD patterns of: a) **44tBubpy** calculated from single crystal data, refcode MUBJOJ, b) **NBSac**, c) product obtained by grinding **44tBubpy** and **NBSac** in a 1:1 stoichiometric ratio for 10 minutes, d) product obtained by dissolving **44tBubpy** and **NBSac** in a 1:1 stoichiometric ratio in dichloromethane.


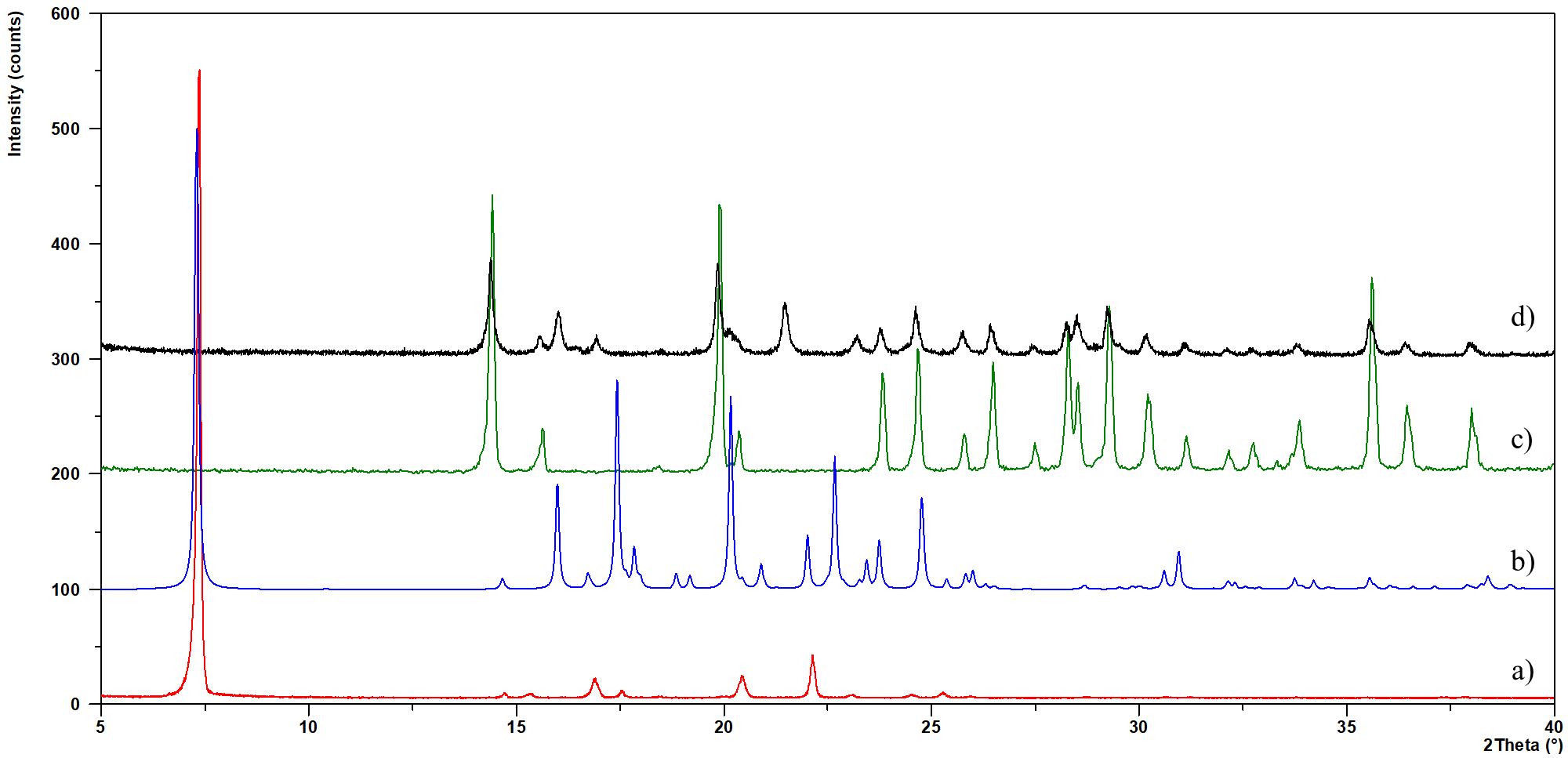


**Figure S24.** PXRD patterns of: a) **44tBubpy** calculated from single crystal data, refcode MUBJOJ, b) **44tBubpy**, reactant powder pattern, c) **NBS**, d) product obtained by grinding **44tBubpy** and **NBS** in a 1:1 stoichiometric ratio.


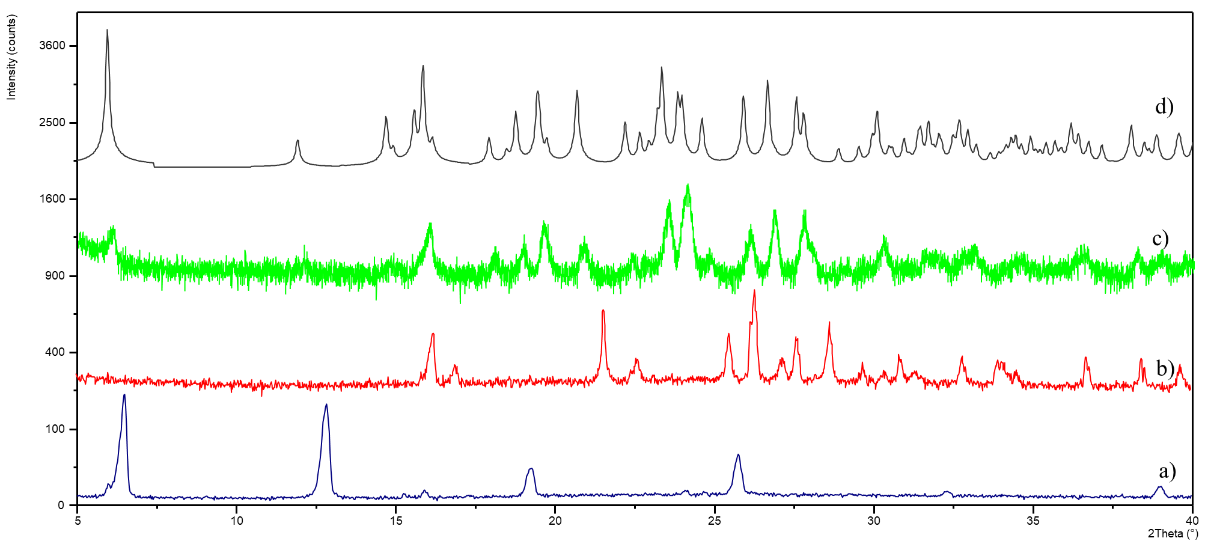


**Figure S25.** PXRD patterns of: a) **22biq**, b) **14tfib**, c) product obtained by grinding **22biq** and **14tfib** in a 1:1 stoichiometric ratio d) calculated pattern from single crystal data.


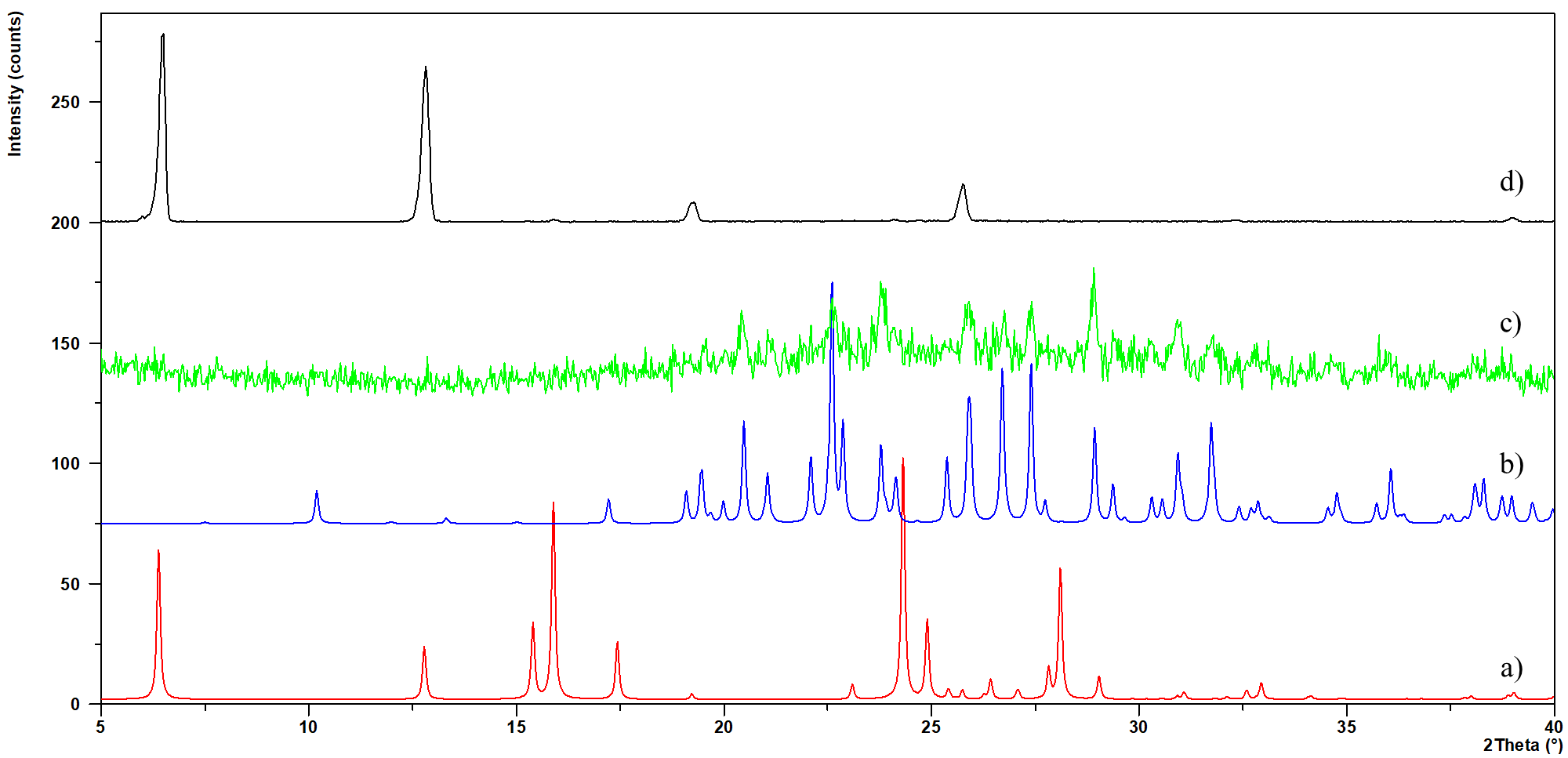


**Figure S26.** PXRD patterns of: a) **22biq** calculated from single crystal data, b) **135tfib** calculated from single crystal data, c) product obtained by grinding **22biq** and **135tfib** in a 1:1 stoichiometric ratio d) product obtained by grinding **22biq** and **135tfib** in a 2:1 stoichiometric ratio.


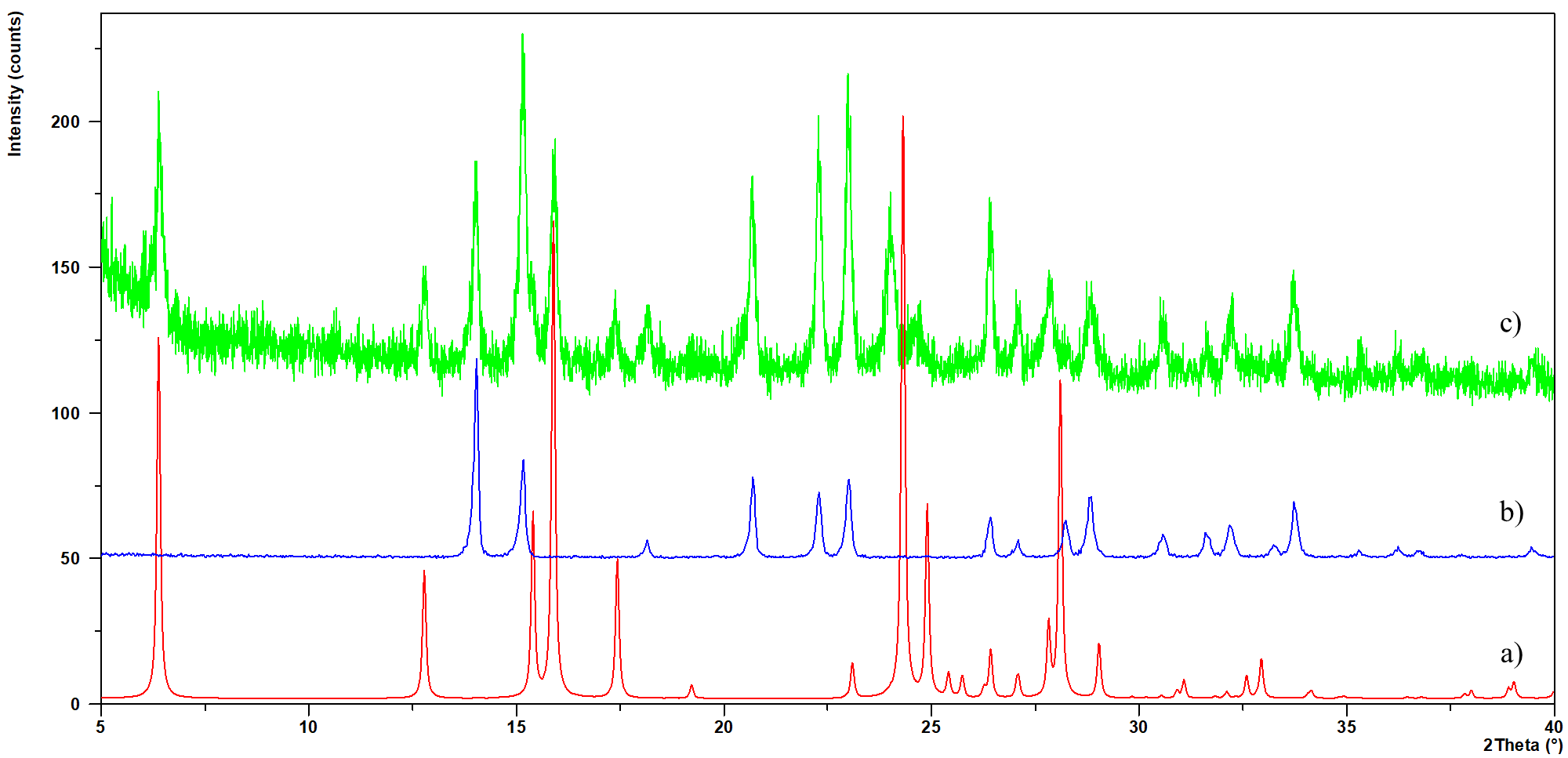


**Figure S27.** PXRD patterns of: a) **22biq** calculated from single crystal data, b) **NIS**, c) product obtained by grinding **22biq** and **NIS** in a 1:1 stoichiometric ratio.


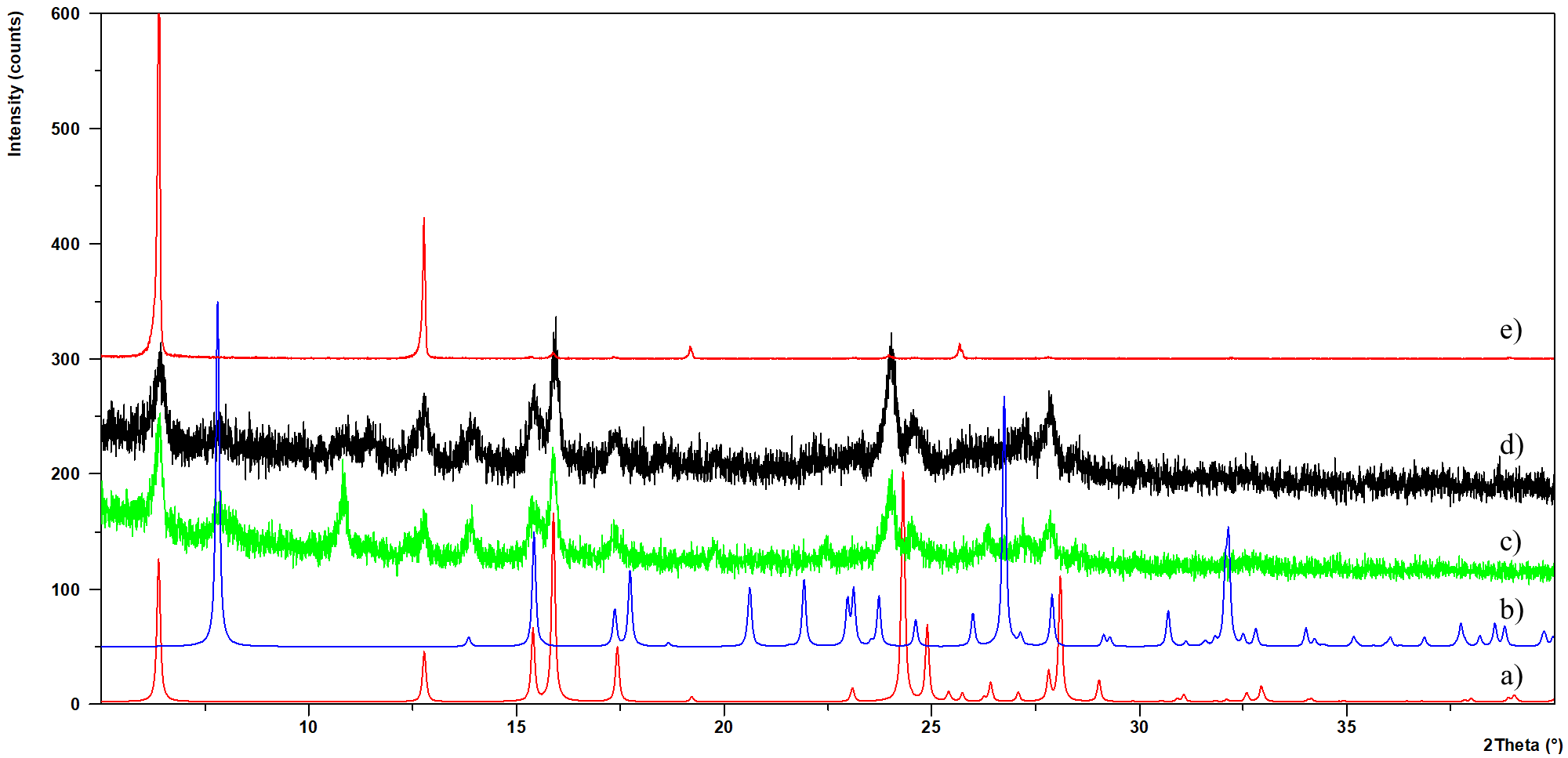


**Figure S28.** PXRD patterns of: a) **22biq** calculated from single crystal data, b) **NBF**, c) product obtained by grinding **22biq** and **NBF** for 10 minutes in a 1:1 stoichiometric ratio, d) product obtained by grinding **22biq** and **NBF** for 30 minutes in a 1:1 stoichiometric ratio, e) crystallization bulk obtained by dissolving **22biq** and **NBF** in acetonitrile in a 1:1 stoichiometric ratio and letting the solvent evaporate at room temperature.


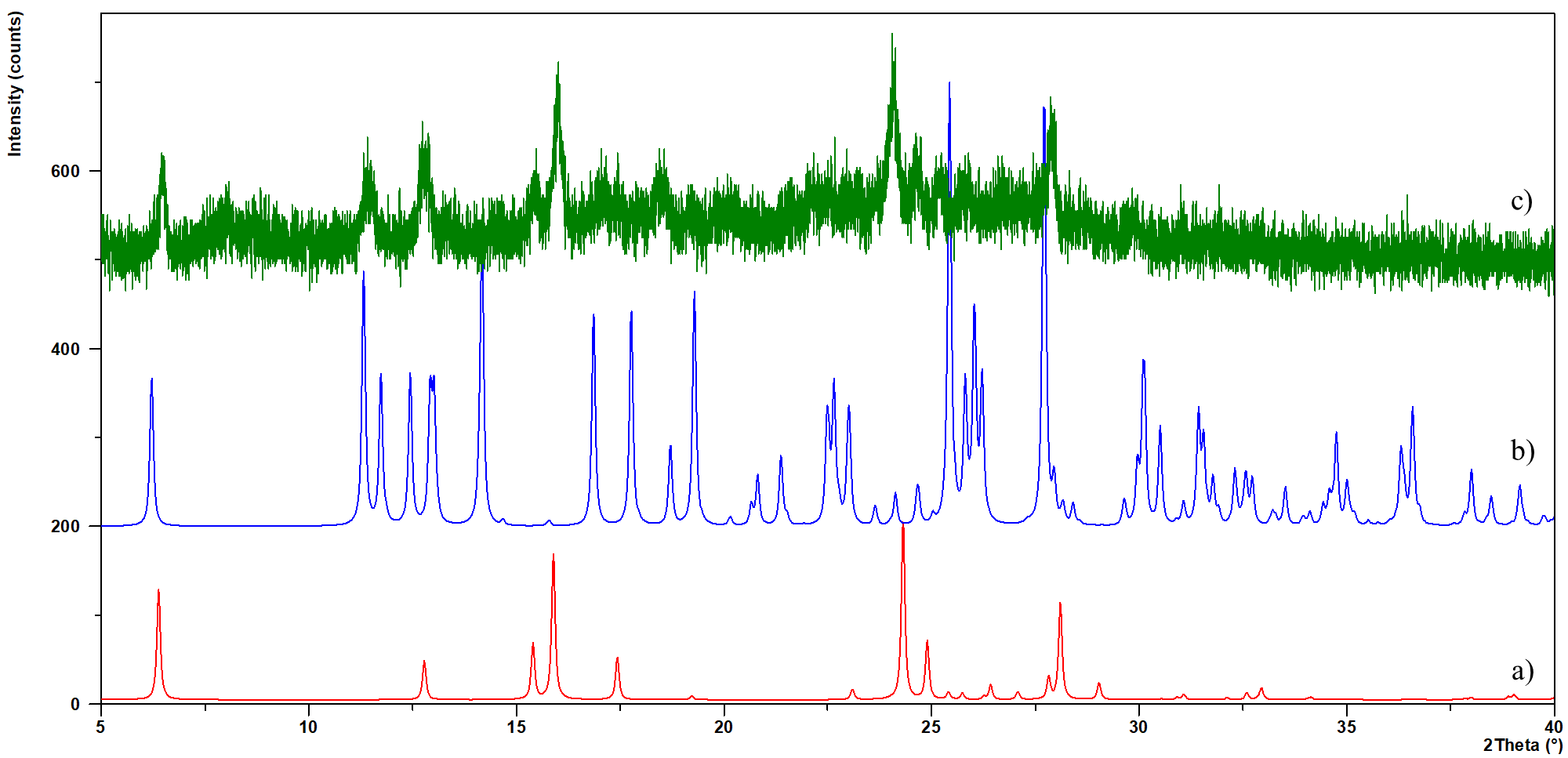


**Figure S29.** PXRD patterns of: a) **22biq** calculated from single crystal data, b) **NBSac**, c) product obtained by grinding **22biq** and **NBSac** in a 1:1 stoichiometric ratio.


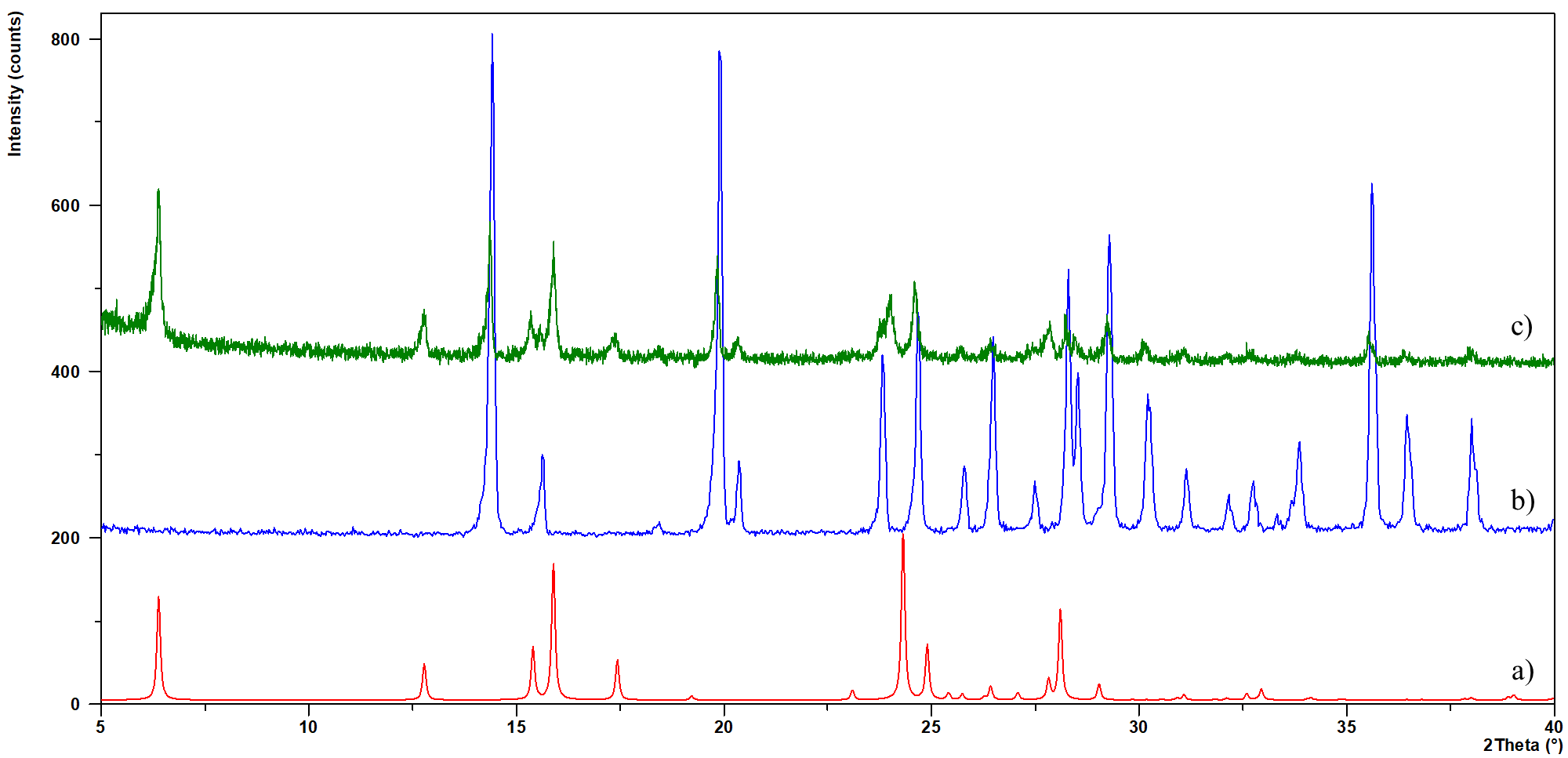


**Figure S30.** PXRD patterns of: a) **22biq** calculated from single crystal data, b) **NBS**, c) product obtained by grinding **22biq** and **NBS** in a 1:1 stoichiometric ratio.


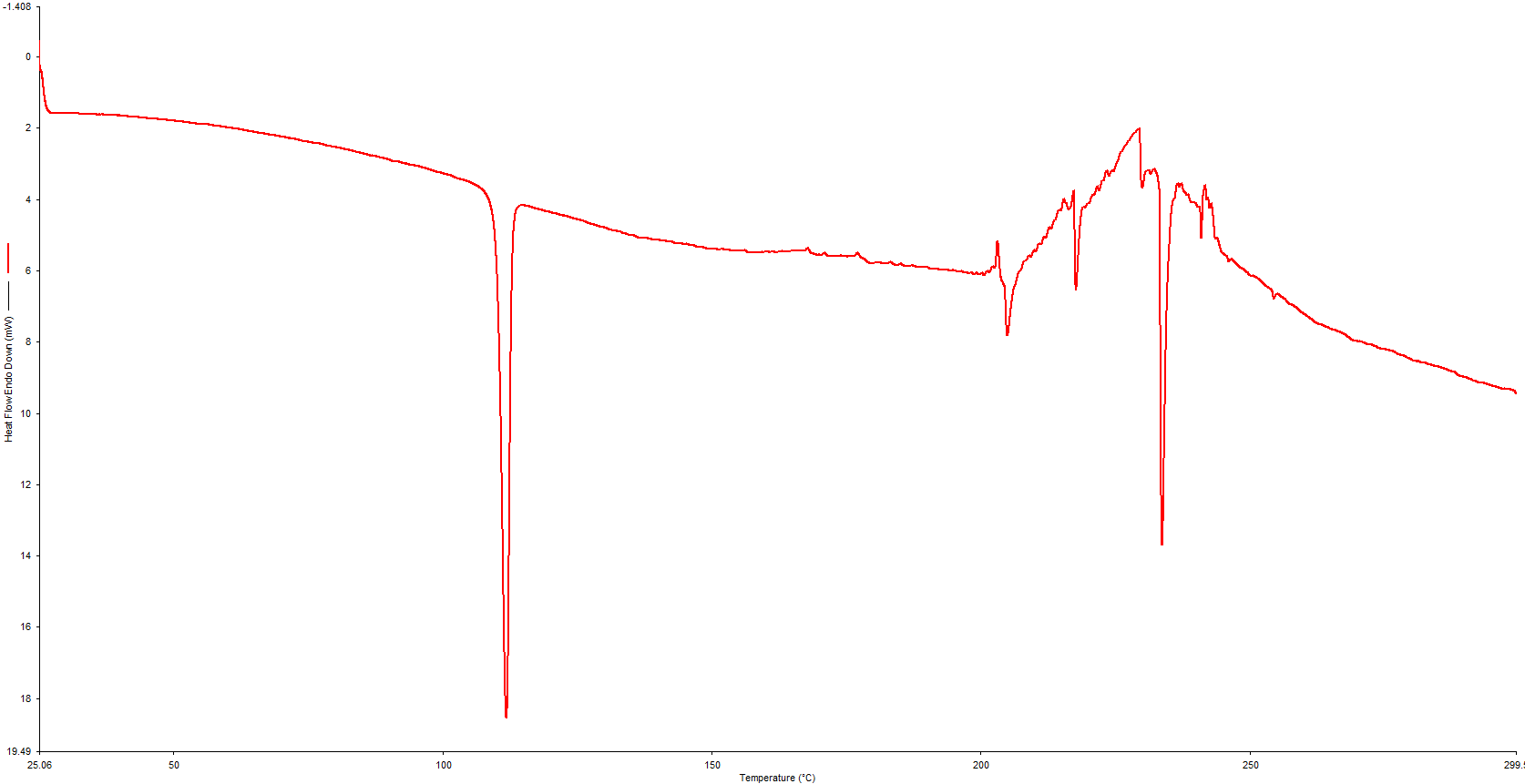


**Figure S31.** DSC curve of (**44diMebpy**)(**135tfib**)_2_.


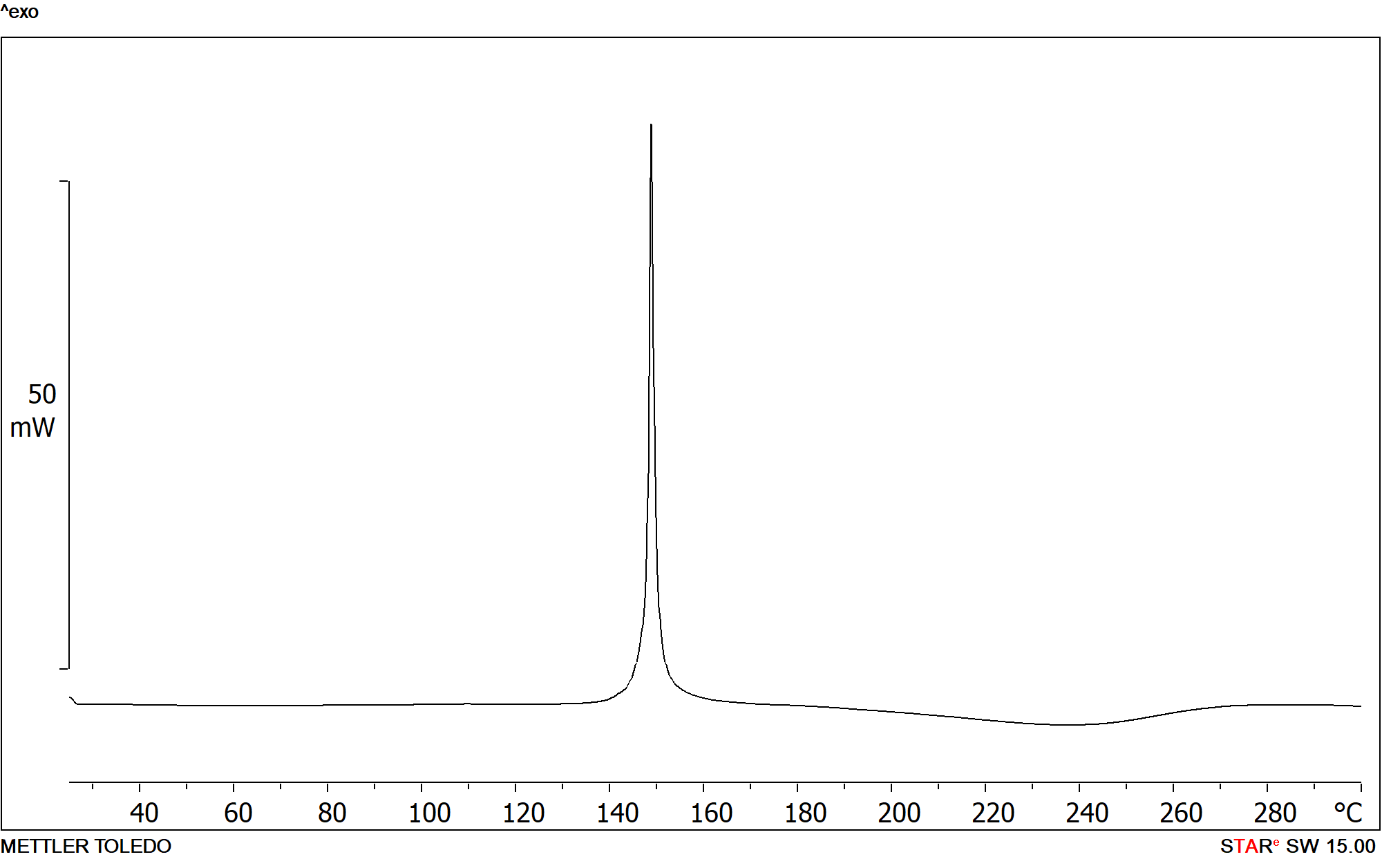


**Figure S32.** DSC curve of (**44diMebpy**)(**NIS**)_2_.


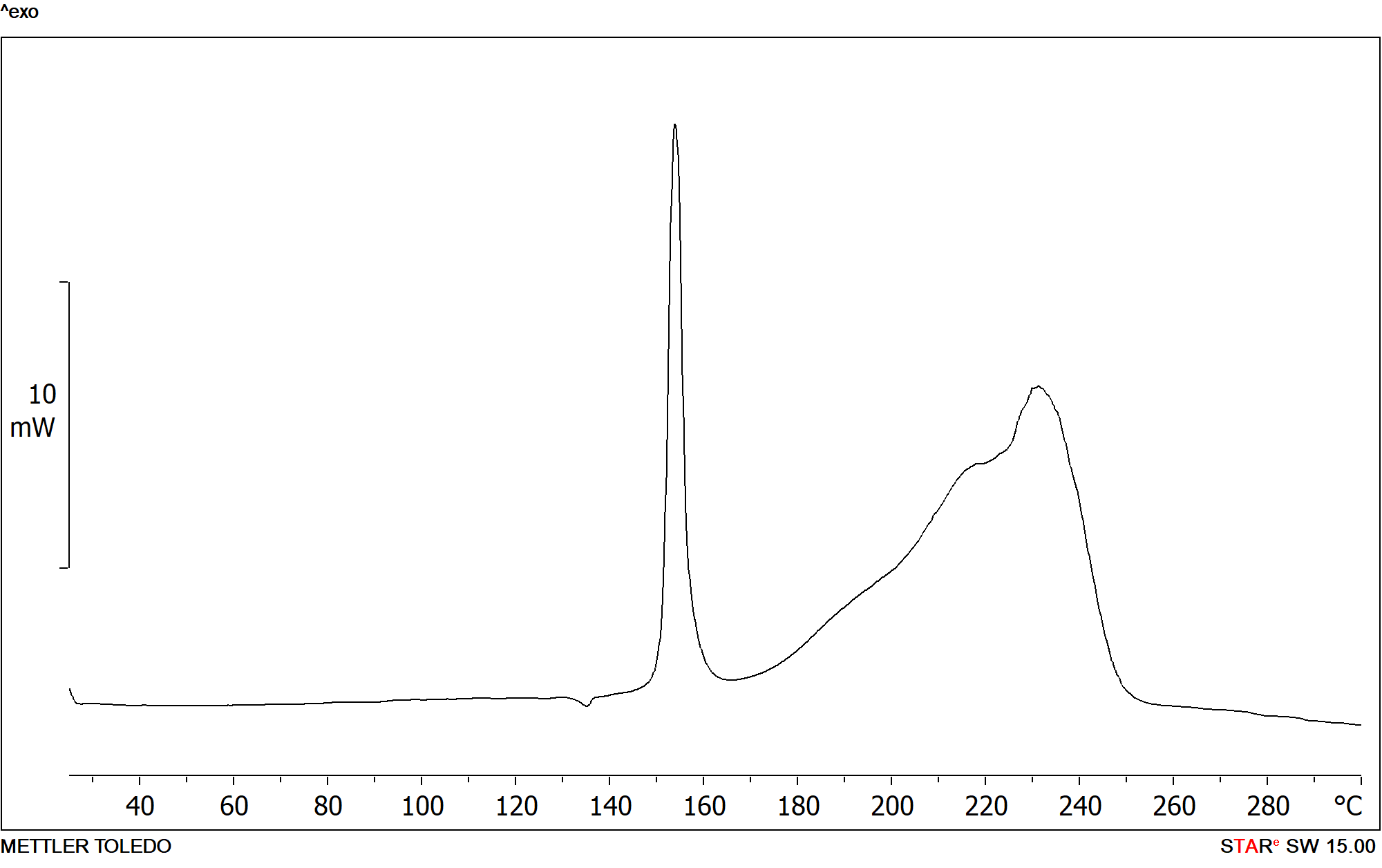


**Figure S33.** DSC curve of (**44diMebpy**)(**NBSac**)_2_.


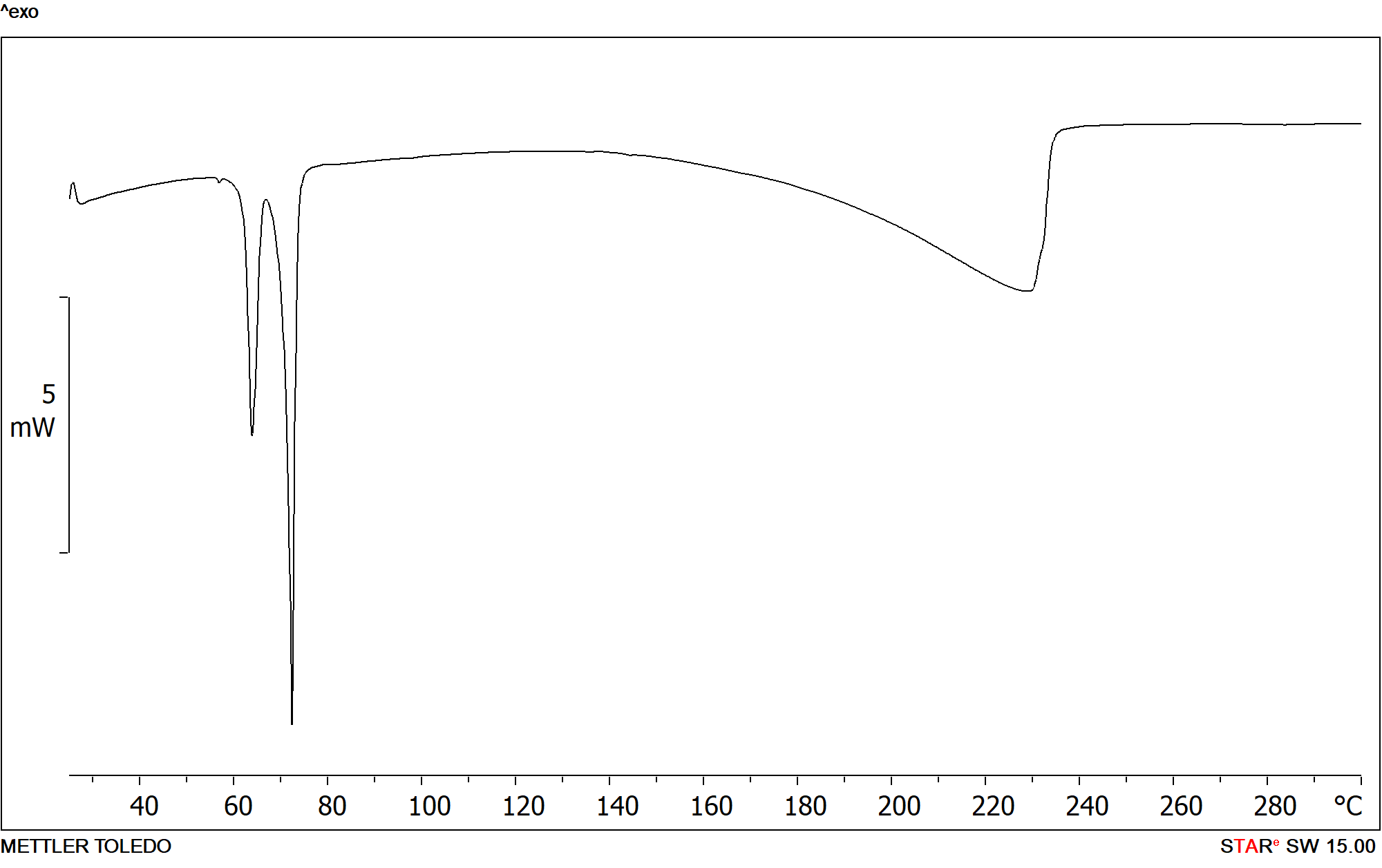


**Figure S34.** DSC curve of (**66diMebpy**)_2_(**44diMebpy**).


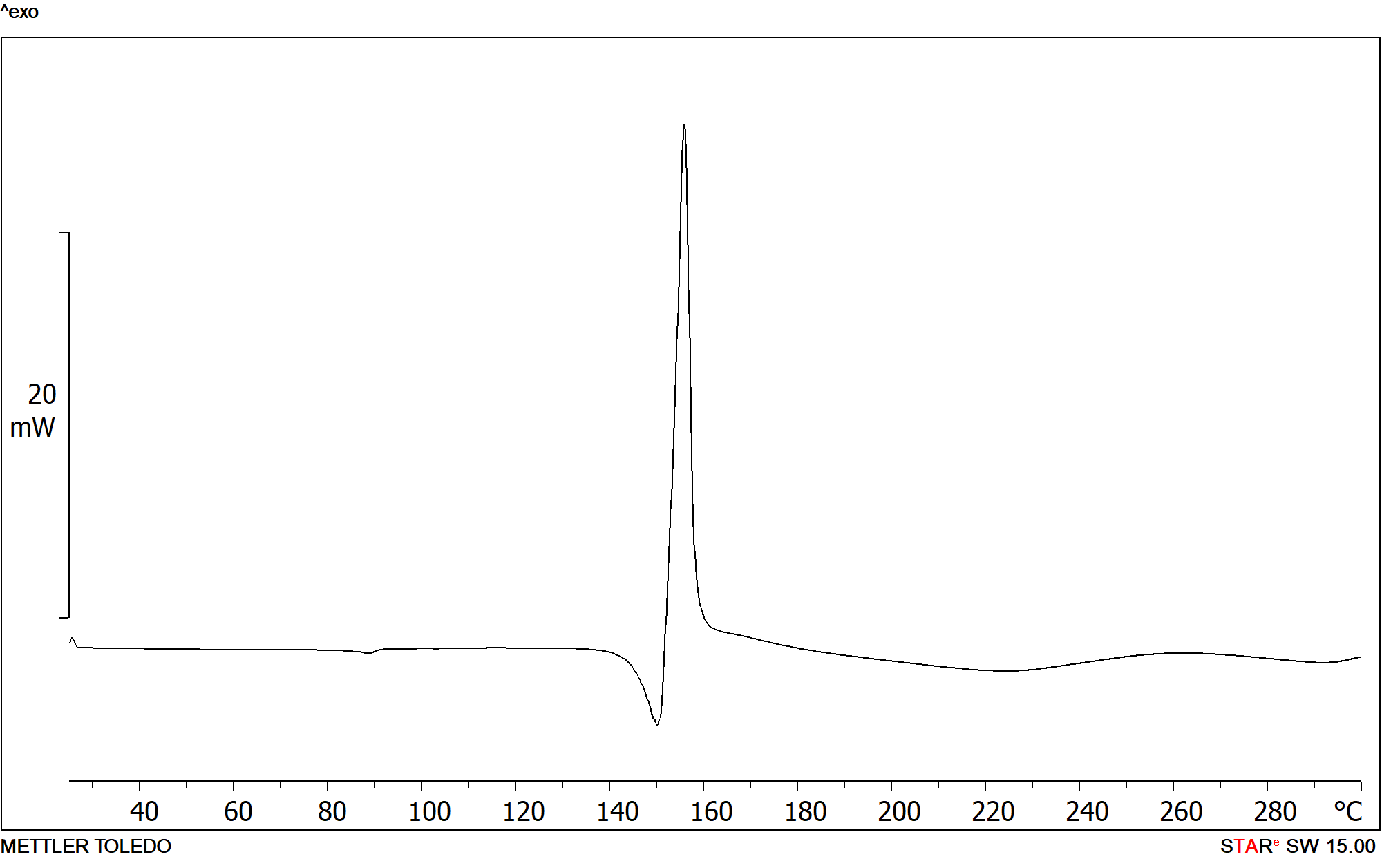


**Figure S35.** DSC curve of (**66diMebpy**)(**NIS**)_2_.


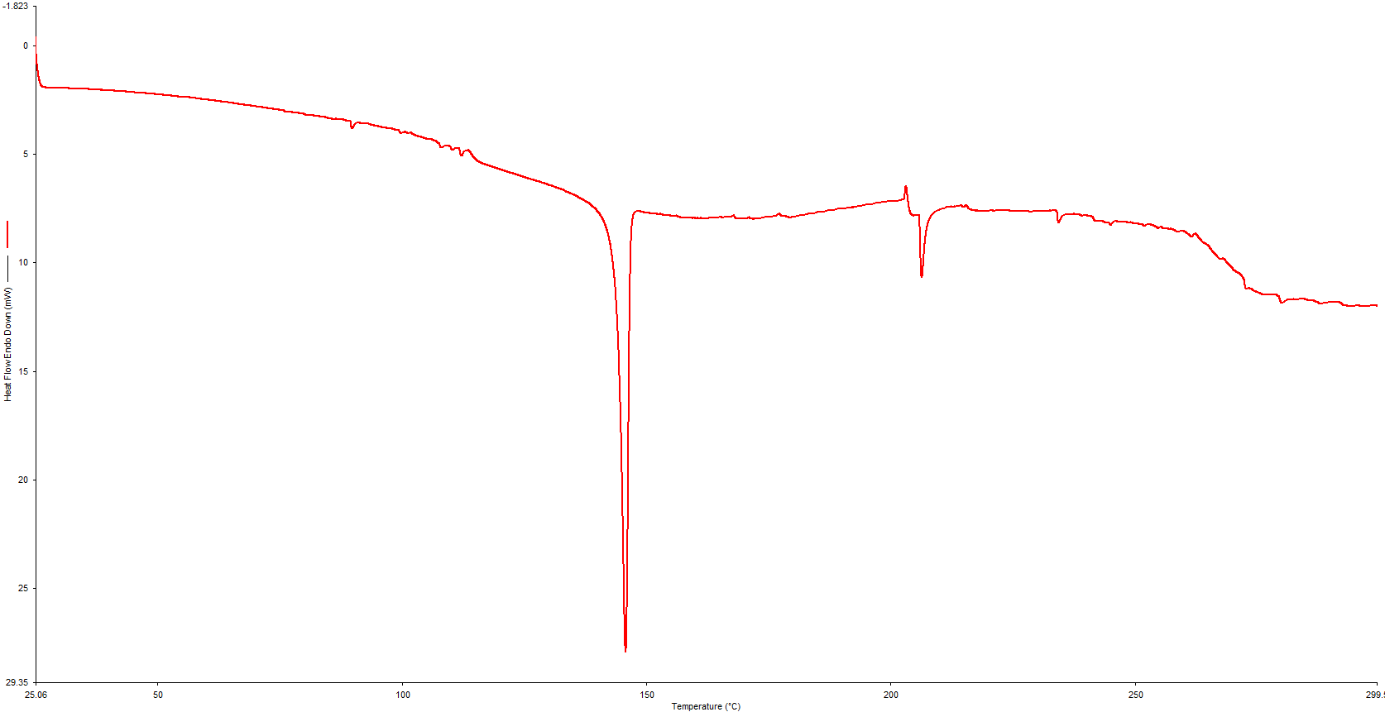


**Figure S36.** DSC curve of (**44tBubpy**)(**14tfib**).


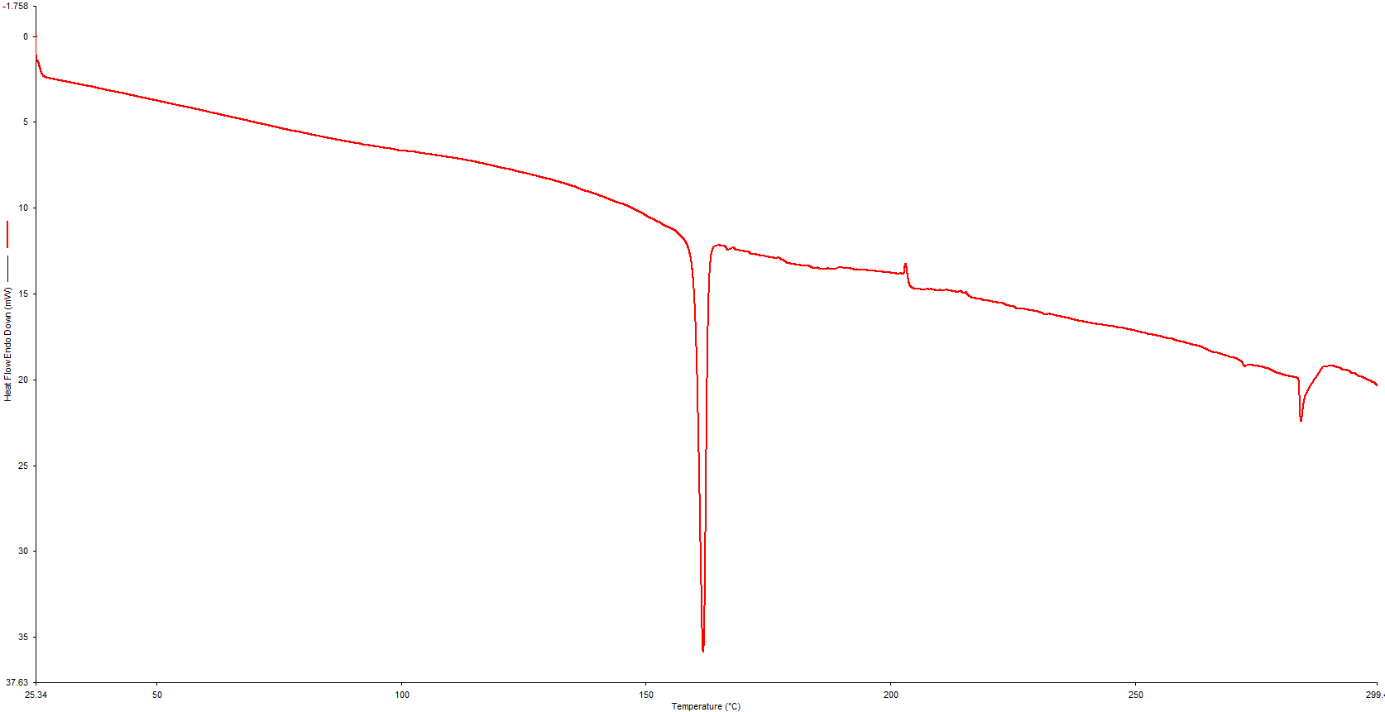


**Figure S37.** DSC curve of (**22biq**)(**14tfib**).
